# The Vaccine Candidates Against Major Fish Pathogens (*Pseudomonas fluorescens* and *Aeromonas hydrophila*) from the Outer Membrane Proteins of *P. fluorescens* in Fish

**DOI:** 10.3390/life16091493

**Published:** 2026-09-06

**Authors:** Xiang Liu, Wei Sun, Ling Zhu, Yuhang Zhan, Yixin Yu, Kai Wang, Qinkai Hu, Xuan Huang, Juan Lu, Xianjie Liu

**Affiliations:** 1 Anhui Province Key Laboratory of Embryo Development and Reproductive Regulation, Anhui Province Key Laboratory of Environmental Hormone and Reproduction, Fuyang Normal University, Fuyang 236041, China; liuxiang888525@fynu.edu.cn (X.L.); 2025221338@stu.fynu.edu.cn (L.Z.); 2025211324@stu.fynu.edu.cn (Y.Z.); yuyixin@stu.fynu.edu.cn (Y.Y.); 2 Chinese-German Joint Institute for Natural Product Research, College of Biological Science and Engineering, Shaanxi University of Technology, Hanzhong 723000, China; 202112003@fynu.edu.cn; 3 Rural Revitalization Collaborative Technology Service Center of Anhui Province, Fuyang Normal University, Fuyang 236041, China; 2023115612@stu.fynu.edu.cn (K.W.); 2023116158@stu.fynu.edu.cn (Q.H.); 2023116122@stu.fynu.edu.cn (X.H.); 200107024@fynu.edu.cn (J.L.); 4 Shenzhen International Institute for Biomedical Research, Shenzhen 518116, China

**Keywords:** *P. fluorescens*, *A. hydrophila*, outer membrane proteins, vaccines, aquaculture, passive immunization, active immunization

## Abstract

Vaccines have demonstrated greater efficiency in providing immunoprotection against bacterial species, making them potentially valuable in aquaculture. In this study, twenty-four outer membrane proteins (OMPs) of *Pseudomonas fluorescens* were cloned, purified, and 16 OMP mouse antisera were prepared, with titers all exceeding 1:3200. Subsequently, these 16 antisera were used to passively immunize crucian carp (*Carassius auratus*) followed by challenge with the pathogenic bacteria. The results showed that five OMP antisera (PF0542, SurA, PF1798, PF2253, and PF4616), as well as the whole OMP serum, provided immune protection rates exceeding 60% against *P. fluorescens* and *Aeromonas hydrophila* infection (*p* < 0.05). Moreover, these five OMP antisera reduced the mRNA expression of inflammatory cytokines and antioxidant factors (*p* < 0.05), and exerted protective effects on the structural integrity of the kidney, spleen, and intestinal tissues. In addition, active immunization of crucian carp with these five identified OMPs followed by pathogen challenge demonstrated that these proteins could activate non-specific immune responses in fish, confer immune protection against *P. fluorescens* and *A. hydrophila* infection, reduce the mRNA expression of inflammatory cytokines and antioxidant factors (*p* < 0.05), and protect the tissue structure of the kidney, spleen, and intestine. Collectively, these five OMPs (PF0542, SurA, PF1798, PF2253, and PF4616) can activate immune responses in crucian carp, confer protection against bacterial infection, and exhibit reductions in inflammatory/oxidative responses associated with protection following bacterial challenge, and as well as viscera structure-maintaining effects. Therefore, the five OMPs hold promise as vaccine candidates against bacterial infections (*P. fluorescens* and *A. hydrophila*) for both passive and active immunization in fish.

## 1. Introduction

The fish farming industry plays a crucial role in providing people with rich protein nutrition and generating substantial economic benefits [1]. However, pathogenic bacterial infections pose a significant threat to fishery cultivation, leading to substantial economic losses each year. This issue has a profound impact on the development of fishery cultivation [2,3,4]. One of the major pathogens in fish farming is *Pseudomonas fluorescens*, a Gram-negative bacterium belonging to the genus *Pseudomonas*. *P. fluorescens* is a rod-shaped flagellate that secretes yellow–green fluorescent pigment to emit fluorescence. The bacterium can infect commonly cultured fish species, such as crucian carp, perch, and carp, and is a major pathogen to aquaculture [5,6]. The infection of fish by *P. fluorescens* manifests with various symptoms, including inflammation of the fish body surface, loss of scales, congestion and erosion of fins, and red skin disease [7,8]. Furthermore, *P. fluorescens* is not only a pathogen for fish but also a zoonosis, posing a risk to other animals and humans. It is also considered a harmful microorganism in milk [9,10]. In addition, several other pathogenic bacteria are found in aquaculture, such as *Aeromonas hydrophila*, *Vibrio fluvialis*, *Vibrio alginolyticus*, *Vibrio parahaemolyticus*, and *Edwardsiella* [8,11]. *A. hydrophila* is a ubiquitous opportunistic pathogen found in freshwater, sewage, and soil, with a broad host range encompassing humans, animals, and fish [12]. It serves as the primary causative agent of bacterial septicemia in freshwater fish. Characterized by a rapid disease onset of 3 to 5 days, this pathogen can induce mass mortality in fish populations, thereby imposing substantial economic losses on aquaculture operations [13]. These pathogens cause significant losses in the aquaculture industry and present challenges in disease prevention and control.

Currently, the use of antibiotics is the primary approach for addressing *P. fluorescens*, but their excessive use can lead to the emergence of drug-resistant bacteria. Moreover, antibiotics hinder the phagocytic function of phagocytes, resulting in reduced fish immunity [14,15]. It also gives rise to additional concerns, such as drug residues and environmental contamination [16,17]. In the realm of pathogen control research, the efficacy of Chinese herbal medicine is gradual, requiring further investigation into its control mechanisms. Additionally, such a vaccine is still in its nascent stage of research and is rarely utilized in aquaculture [18]. Hence, the development of novel drugs is imperative. Among them, the recombinant protein subunit vaccine stands out as an immunologically active fragment generated through genetic engineering. In particular, polyvalent protein vaccines exhibit the potential to safeguard against multiple bacterial infections, boasting the advantages of both safety and efficacy [19]. The study of polyvalent protein vaccines represents a thriving domain within vaccine research.

Outer membrane proteins (OMPs) exist on the surface of Gram-negative bacteria. These OMPs account for approximately half of the entire outer membrane area and play crucial roles in maintaining cell structure, transmitting information, transporting materials, and resisting antibiotics [20,21]. By directly interacting with the host, OMPs mediate the interaction between bacteria and the host, thereby influencing bacterial infection and the host’s resistance to it [22,23]. Recent research has indicated that OMPs possess strong immune activity due to their direct contact with the host [24,25]. One notable example is the outer membrane protein F of *P. fluorescens*, which can activate fish-specific immunity and effectively guard against *P. fluorescens* infection in animals [26]. Another protein, the TonB-dependent outer membrane receptor (TdrA) of *P. fluorescens*, consists of 746 residues and has the ability to stimulate the production of a specific antiserum that protects fish against *P. fluorescens* infection [27]. In an immunization study conducted on turbot, Tdr1, Tdr2, and Tdr3 were found to activate immune responses to resist *P. fluorescens* infection [26]. Additionally, the immune-protective effects of OMPs from other bacterial species, such as OmpA, OmpC, and OmpK, have been documented [28,29,30,31]. Consequently, there is a need for extensive screening and identification of the OMPs in *P. fluorescens*, particularly those with cross-protective abilities against different bacterial species.

**Figure 1:**
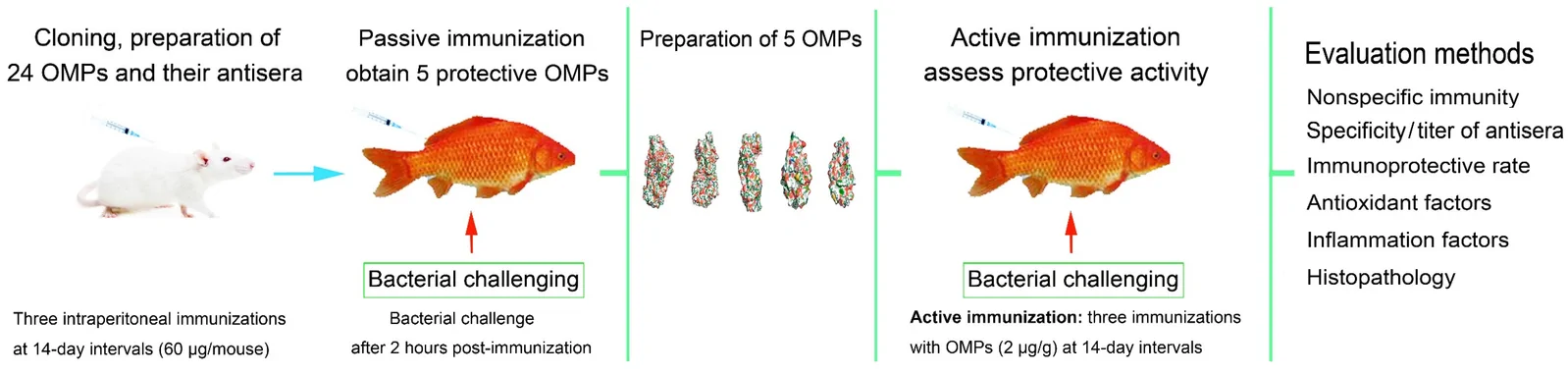
Experimental design route.

In this study, we utilized 24 known OMPs of *P. fluorescens* ATCC 13525 from the database of National Center for Biotechnology Information (NCBI) to evaluate their immunogenic activities and explore their potential for vaccine applications. Then, we successfully cloned 24 OMPs from *P. fluorescens*. The immune capability of these OMPs was initially assessed by conducting passive immune evaluation experiments in crucian carp (*Carassius auratus*). Their immune function was further determined through an active immunization experiment in *C. auratus*, thereby establishing a solid foundation for the development of an active immunization vaccine (Figure 1). This comprehensive investigation aims to provide a fundamental framework for the research of protective vaccines in aquaculture.

## 2. Materials and Methods

### 2.1. Bacterial Strains and Animals

*P. fluorescens* ATCC 13525, *A. hydrophila* ATCC 35654, and pET-32a plasmid were stored in our microbiology laboratory. *Escherichia coli* BL21 and *E. coli* DH5a competent cells were purchased from Solebao Biotechnology Co., Ltd. (Beijing, China). *C. auratus* (15 ± 1.5 g), Leghorn laying hens (20 weeks old), and Kunming mice (20 ± 1.5 g) were procured from Green Aquaculture Co., Ltd. (Fuyang, China), Anhui Tengxin Animal Breeding Co. Ltd. (Hefei, China), and Anhui Medical University (Hefei, China), respectively. Mouse experiments were conducted at the Animal Husbandry Center of Fuyang Normal University from 15 May 2023 to 15 July 2023. Fish experiments were conducted at the Aquaculture Laboratory of Fuyang Normal University from 30 August 2023 to 30 December 2023. *C. auratus* were acclimatized to laboratory conditions for 14 days prior to the start of the immunization experiments. The passive immunization and challenge experiments of fish were completed within the first 8 weeks, followed by the active immunization and challenge experiments over the subsequent 10 weeks. All animal procedures were performed in accordance with the guidelines prescribed in the Guide for the Care and Use of Laboratory Animals and approved by the Institutional Animal Care and Use Committee of Fuyang Normal University, China (Approval No: 2023-0515, 16 April 2023).

### 2.2. The Preparation of Entire OMPs

5 mL of an overnight culture of *P. fluorescens* was inoculated into 600 mL of LB medium and cultured at 30 °C for 5 h. The cells were harvested by centrifugation at 4 °C and 4000 r/min for 10 min. After washing three times with normal saline, the pellet was resuspended in 30 mL of phosphate-buffered saline (PBS) solution. The cells were disrupted using an ultrasonic cell disruptor for 10 min. Following centrifugation at 4000 r/min for 10 min, the supernatant was collected and centrifuged at 23,000 r/min for 30 min. Then, the pellet was resuspended in 2 mL of 20 mmol/L Tris-HCl (pH 7.6, containing 2% Sarkosyl). After incubation in a 32 °C water bath for 30 min, the solution was centrifuged at 23,000 r/min for 30 min. Finally, the pellet was resuspended in 20 mmol/L PBS solution to obtain the entire OMPs, and stored in a −80 °C freezer [32].

### 2.3. Cloning and Purification of P. fluorescens OMPs

Twenty-four OMP gene sequences were obtained from the NCBI database using the genome information of *P. fluorescens* ATCC 13525 (GenBank: LT907842.1). Specific primers were designed by the Primer Premier 5.0 software (Premier Biosoft, Palo Alto, CA, USA) for the OMP genes (Table 1). PCR was performed using the genomic DNA of *P. fluorescens* extracted using a genome kit (Sangon Biotech Co., Ltd., Shanghai, China). The pET-32a plasmid vector and 24 OMP genes were digested with the corresponding endonuclease, ligated overnight using T4-DNA ligase (Sangon Biotech Co., Ltd., Shanghai, China), and transferred into an *E. coli* DH5a strain. The OMP plasmids were extracted from the *E. coli* DH5a strain using a plasmid extraction kit (TAKARA, Dalian, China) and verified using endonuclease double digestion and sequencing (Sangon Biotech Co., Ltd., Shanghai, China). Subsequently, the OMP plasmids were transformed into *E. coli* BL21 to establish a recombinant protein expression strain.

The expression and purification of OMPs were performed according to previously published methods [33]. Briefly, an overnight culture of the OMP-expressing strain was transferred into 600 mL of fresh LB medium. The OMP recombinant strain was grown until they reached an optical density (*OD*_600_) of 0.8. Subsequently, they were induced for 36 h with IPTG (0.3 mmol/L) (TAKARA, Dalian, China) at 25 °C. To evaluate the expressions of the OMPs, 500 μL of the bacterial fluid was collected and combined with 400 μL of 2× SDS loading buffer. This mixture was subjected to a 5 min boiling process in water, and 10 μL of the solution was taken for SDS-PAGE (sodium dodecyl sulfate polyacrylamide gel electrophoresis) protein electrophoresis. Then, after confirming OMP expression, the remaining bacterial liquid (approximately 600 mL) was collected by centrifugation and disrupted using an ultrasonic cell disruptor. After centrifugation at 10,000 rpm for 10 min, the supernatant containing OMPs was collected, and Ni-NTA flow resin (TAKARA, Beijing, China) was utilized to purify the recombinant OMPs according to the manufacturer’s instructions. Endotoxin was removed from the purified OMPs using Detoxi-Gel™ Endotoxin Removing Columns (Thermo Fisher Scientific, Waltham, MA, USA). The pET-32a vector-derived tag protein, which served as a control, was subjected to the same treatment to exclude any potential confounding effects of residual lipopolysaccharide. Finally, the purity of the OMPs was assessed by SDS-PAGE protein electrophoresis, and the concentration of the OMPs was determined using a BCA (bicinchoninic acid) protein assay kit (TAKARA, Dalian, China).

### 2.4. Preparation and Identification of OMP Antibodies

The Kunming mice were immunized intraperitoneally with 24 OMPs. For each OMP, eight mice were immunized intraperitoneally at a dosage of 60 μg per mouse. To enhance the immunization process, Freund’s complete adjuvant (Sangon Biotech Co., Ltd., Shanghai, China) was used as an emulsifier in the first immunization. Subsequently, Freund’s incomplete adjuvant was employed during the second and third immunizations to further strengthen the immune response. The mice of negative control (NC) were immunized with vector tag protein of pET-32a plasmid. The duration of each immunization was 14 days. The mice were anesthetized, and their blood was collected from the eyeballs after the third immunization under anesthesia. The OMP antiserum was then acquired through centrifugation at 3000 r/min for 15 min [34].

The specificity and titer of the OMP mouse antibodies were evaluated with Western blotting [34]. Briefly, *P. fluorescens* was cultured overnight and prepared for SDS-PAGE protein electrophoresis. Subsequently, SDS-PAGE gel was transferred to the nitrocellulose membrane at 80 V for 1.5 h by a membrane transfer instrument. The nitrocellulose membrane was incubated in a 5% skim milk solution. Afterward, the OMP sera were applied to the nitrocellulose membrane at varying dilutions and incubated for 1 h at 37 °C. To remove any unbound antibodies, the nitrocellulose membrane was washed three times with PBS solution. Next, secondary antibodies of goat anti-mouse IgG (1:3000) (Sigma-Aldrich, St. Louis, MO, USA) were applied to the nitrocellulose membrane. Finally, a diaminobenzidine substrate system was used to visualize the protein bands on the nitrocellulose membrane.

### 2.5. Evaluation of the Passive Immunoprotective Rate of OMP Antibodies in C. auratus

The challenge doses of *P. fluorescens* and *A. hydrophila* in fish were determined by preliminary dose–response experiments [19]. In brief, groups of *C. auratus* (n = 10 per group) were intraperitoneally administered 40 μL of blank serum. At 2 h post-administration, the fish were challenged with increasing doses of *P. fluorescens* (1.0, 2.0, 3.0, and 4.0 × 10^9^ CFU) and *A. hydrophila* (3.0, 4.0, 5.0, and 6.0 × 10^9^ CFU), and mortality was observed over a 4-day period. The values of median lethal dose (LD_50_) for *P. fluorescens* and *A. hydrophila* were calculated as 3.0 × 10^9^ CFU and 4.0 × 10^9^ CFU, respectively. These LD_50_ values were adopted as the challenge doses for all subsequent experiments.

To evaluate the passive immunoprotective rate, *C. auratus* was subjected to immunization with OMP antibodies and infected with bacteria, as described by Xiao et al. [19]. In brief, *P. fluorescens* and *A. hydrophila* were routinely cultured in Luria–Bertani (LB) broth (containing 10 g/L tryptone, 5 g/L yeast extract, and 10 g/L NaCl, pH 7.0) at 30 °C with shaking at 200 r/min. Overnight cultures were inoculated into fresh LB medium at a 1:100 dilution and grown to an optical density at 600 nm (*OD*_600_) of 1.0 for subsequent pathogen challenge experiments. Then, each group of *C. auratus* (15 fish) was intraperitoneally immunized with OMP mouse antibodies at different amounts based on their titer, as detailed in Table 2. The negative control (NC) group received a blank serum, which was obtained by immunizing mice with vector tag protein of pET-32a plasmid. After a 2 h interval with antibodies, *C. auratus* were intraperitoneally challenged to either *P. fluorescens* (3.0 × 10^9^ CFU) or *A. hydrophila* (4.0 × 10^9^ CFU) with 40 μL per fish according to the preliminary experiment. The death situation of the *C. auratus* individuals was observed for 14 days. Subsequently, the relative percent survival (RPS) of *C. auratus* was calculated as follows: RPS (%) = [1 − (% OMPs mortality/% control mortality)] × 100. SPSS 19.0 software was employed to evaluate significant differences between the OMP and NC groups.

After the completion of the challenge experiments, all materials contaminated with live *P. fluorescens* and *A. hydrophila* cultures were decontaminated prior to disposal [33,34]. Briefly, solid waste (e.g., culture plates, pipette tips, and gloves) and liquid waste (e.g., spent culture media and bacterial suspensions) were collected and autoclaved at 121 °C for 30 min before disposal as non-hazardous waste. Fish tissues and carcasses were collected and incinerated by Fuyang Waste Disposal Co., Ltd. (Fuyang, China) in accordance with institutional and national biosafety regulations.

### 2.6. The Innate Immune Abilities of OMPs

The *C. auratus* were administered intraperitoneal immunization of OMPs (2 μg/g), while the negative control group (NC) was given pET-32a plasmid tag protein. Each group consisted of six fish. The fish were immunized with OMPs three times at an interval of 14 days with Freund’s incomplete adjuvant as an emulsifier. OMP antisera were obtained from the caudal vein of the fish after the third immunization under anesthesia. The levels of lysozyme (LZM), immunoglobulin M (IgM), alkaline phosphatase (AKP), and acid phosphatase (ACP) were measured in the serum according to the kit instructions (Sangon Biotech Co., Ltd., Shanghai, China). The experiment was performed in triplicate (n = 3) [33].

### 2.7. Active Immunoprotective Rate of OMPs

The *C. auratus* were administered three intraperitoneal immunizations to OMPs, with a dosage of 2 μg/g for each fish. The negative control group (NC) was immunized to vector tag protein of pET-32a plasmid with a dosage of 2 μg/g for each fish. The immunization schedule included a 14-day interval between each vaccination. The OMPs were emulsified with Freund’s incomplete adjuvant in each immunization process. Following the third immunization, the *C. auratus* were intraperitoneally challenged with either *P. fluorescens* (3.0 × 10^9^ CFU) or *A. hydrophila* (4.0 × 10^9^ CFU) with 40 μL per fish according to the preliminary experiment. To assess mortality, the *C. auratus* were fed for 14 days to assess the RPS. SPSS 19.0 software was employed to evaluate the significant differences between the OMP and NC groups [33].

### 2.8. Antioxidant Factors of C. auratus

On day 2 following the bacterial challenge, serum samples were obtained from the caudal vein of the fish. Each group consisted of six fish. The antioxidant-related factors of catalase (CAT), superoxide dismutase (SOD), glutathione peroxidase (GSH-Px), and malondialdehyde (MDA) were evaluated according to the instructions of the detection kit (Sangon Biotechnology Co., Ltd., Shanghai, China). The experiment was performed in triplicate (n = 3).

### 2.9. mRNA Expression of Inflammation Factors in C. auratus

The real-time quantitative PCR (qRT-PCR) method was performed to assess the mRNA expression of inflammatory factors [19]. Briefly, on day 2 post-bacterial challenge, tissue samples (gill, kidney, and spleen) were aseptically collected from three fish per group (PF0542, SurA, PF1798, PF2253, PF4616, and the control group). The tissues were immediately frozen in liquid nitrogen and stored at −80 °C until RNA extraction. Total RNA was isolated from approximately 50 mg of each tissue sample using TRIzol^®^ reagent (Takara, Dalian, China) according to the manufacturer’s instructions. The concentration and purity of the extracted RNA were determined spectrophotometrically by measuring the absorbance at 260 nm and 280 nm (NanoDrop™ 2000, Thermo Fisher Scientific, Waltham, MA, USA). Only RNA samples with an A260/A280 ratio between 1.8 and 2.0 were used for subsequent cDNA synthesis. RNA integrity was further verified by electrophoresis on a 1.5% (*w*/*v*) agarose gel, where the presence of clear 28S and 18S rRNA bands indicated intact RNA. Then, the first-strand cDNA was synthesized from 1 μg of total RNA using the PrimeScript™ RT Reagent Kit (Takara, Dalian, China) following the manufacturer’s protocol. The reverse transcription reaction was carried out in a 20 μL total volume containing the above reaction mixture, 1× PrimeScript Buffer, 0.5 mmol/L dNTP, 2.5 μmol/L oligo-dT primer, and PrimeScript RT Enzyme Mix. The reaction was incubated at 37 °C for 15 min, followed by 85 °C for 5 s to inactivate the reverse transcriptase. Subsequently, qRT-PCR was performed using the SYBR^®^ Green Premix kit (Takara, Dalian, China) and synthesized primers (Supplementary Table S1). Each 20 μL reaction mixture consisted of 10 μL of 2× SYBR Premix Ex Taq II, 0.4 μL each of forward and reverse primers (10 μmol/L), 0.4 μL of 50× ROX Reference Dye, 2 μL of diluted cDNA template, and 6.8 μL of RNase-free water. The thermal cycling protocol was as follows: initial denaturation at 95 °C for 30 s, followed by 30 cycles of denaturation at 95 °C for 5 s, annealing at 60 °C for 30 s, and extension at 72 °C for 30 s. The ΔCt (cycle threshold change) values of the experimental and control group were obtained by comparing the Ct value of the target gene with that of the reference gene (*gapdh*). The ΔΔCt value was then calculated by comparing the ΔCt values between the experimental and control group. Subsequently, the mRNA expression of inflammatory factors was analyzed using the 2^−ΔΔCt^ formula. Finally, SPSS 19.0 software was used to assess the significant differences. The experiment was performed in triplicate (n = 3).

### 2.10. Histopathology of C. auratus Viscus

On day 2 following the bacterial challenge, visceral organs (kidney, spleen, and intestine) of *C. auratus* were procured and soaked in 10% formaldehyde solution and Davidson’s fixative for 24 h, respectively. Then, the tissues were subjected to gradient ethanol dehydration treatment and xylene transparency treatment. After being embedded with paraffin, the tissues were sliced at a thickness of 4 μm with a paraffin slicer. The slices were placed on a glass slide to dry for 24 h at 37 °C, and hematoxylin and eosin staining was performed on the slices. After xylene transparency, the slices were sealed in neutral balsam and photographed using a microscope (Leica, Wetzlar, Germany) [33].

All sections were coded by an independent technician, and two pathologists performed scoring while blinded to group assignments. Then, re-evaluation of 20 randomly selected sections after two weeks showed >95% agreement, confirming excellent reproducibility. Under microscopic observation, the lesion to kidney, spleen, and intestinal tissue was assessed. Each group consisted of three fish. Based on the severity of lesion in the kidney and spleen, lesion scoring and quantitative pathology were performed using the semi-quantitative pathological evaluation method of Rabb et al. [35]. Briefly, a higher score indicates more severe lesions in the kidney and spleen (maximum score: 4). Scoring criteria: 0, normal kidney and spleen structure without lesions; 1, minimal tissue lesions (<5% of kidney or spleen tissue affected); 2, mild tissue lesions (5–25% of kidney or spleen tissue affected); 3, moderate tissue lesions (25–75% of kidney or spleen tissue affected); 4, severe necrosis (>75% of kidney or spleen tissue necrosis). In addition, according to the degree of intestinal tissue lesions, the double-blind method of Deng et al. [36]. was adopted for lesion scoring. Scoring criteria: 0, intact intestinal villus structure without lesions; 1, mild villous edema of the intestinal tissue, with epithelial lesions confined to the villus tip; 2, mild lesions in the mid-villus region; 3, moderate necrosis of the mid-villus region; 4, severe villous necrosis with loss of the epithelial tissue structure. The experiment was performed in triplicate (n = 3).

### 2.11. The Statistical Analysis

All biological replicates in this study were performed with a minimum of three independent replicates. Significant differences between the experimental group and control group were evaluated using SPSS 19.0 software with one-way analysis of variance (ANOVA) followed by Tukey test. Prior to parametric analysis, the assumptions of normality and homogeneity of variance were evaluated using the Shapiro–Wilk test and Levene’s test, respectively. Survival data of fish were analyzed using the Kaplan–Meier method, and differences in survival distributions were evaluated using the log-rank (Mantel–Cox) test. Results were expressed as mean ± standard deviation (SD). *p* < 0.05 was considered statistically significant [33].

## 3. Results

### 3.1. Passive Immunoprotection of the Entire OMP Mouse Serum in C. auratus

**Figure 2:**
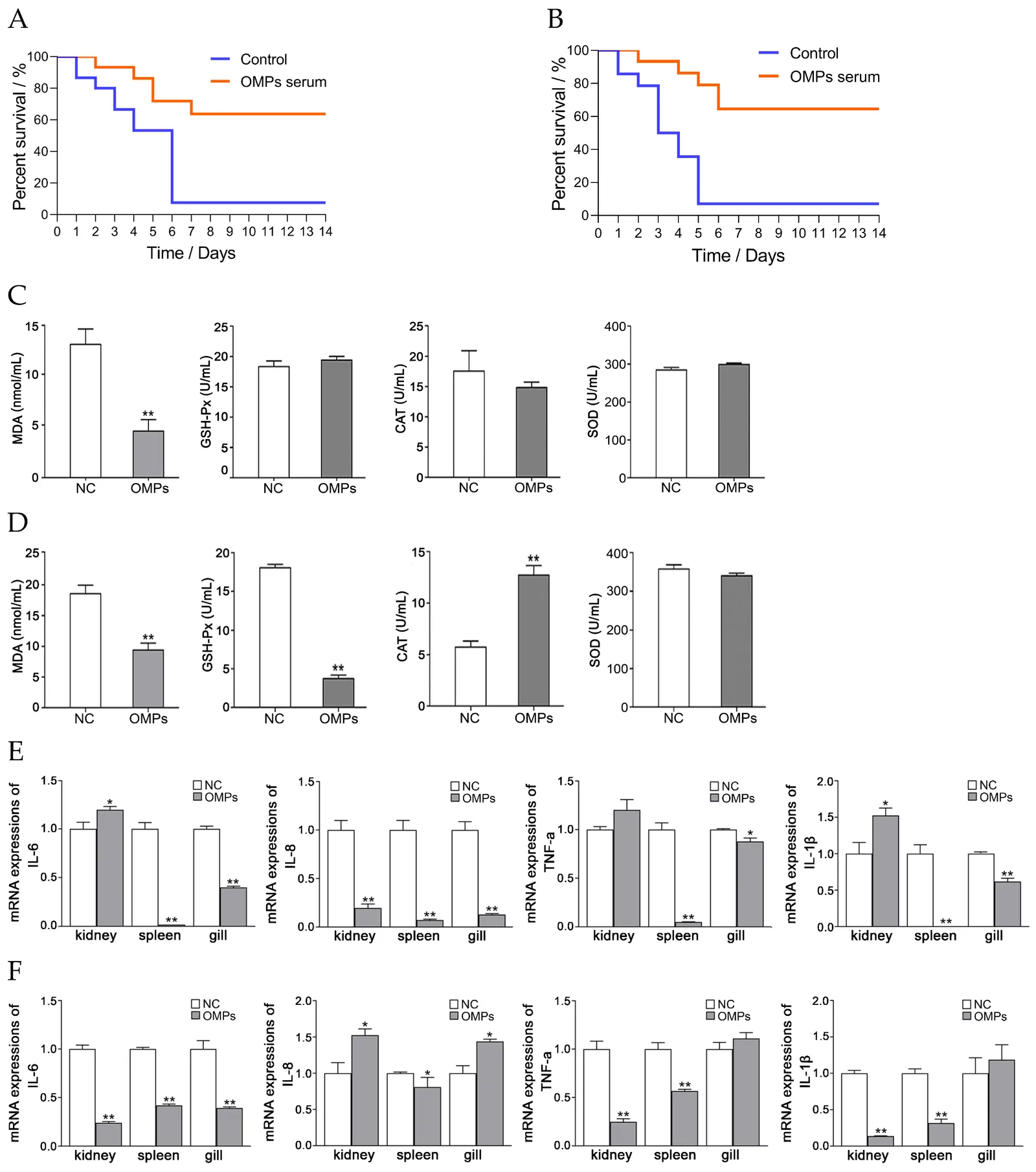
Passive immunoprotection of the entire OMP mouse serum in *C. auratus*. Percent survival of *C. auratus* after being challenged with *P. fluorescens* (**A**) and *A. hydrophila* (**B**). After passive immunization with the entire OMP antiserum and being challenged with *P. fluorescens* (**C**,**E**) and *A. hydrophila* (**D**,**F**) (n = 3). The antioxidant-related factors (MDA, CAT, GSH-Px, and SOD) reduced (**C**,**D**), and the mRNA expressions of inflammation-related genes (*il-6*, *il-8*, *tnf-α*, and *il-1β*) decreased (*p* < 0.05) (**E**,**F**). * *p* < 0.05 and ** *p* < 0.01 (compared with control).

Passive immunization was performed on *C. auratus* using the mouse antiserum of entire OMPs of *P. fluorescens*. The fish were challenged with the bacterium of *P. fluorescens* or *A. hydrophila* to determine the RPS. In the negative control (NC) group (receiving blank serum), high mortality of *C. auratus* was observed in the control group within 6 days. From the seventh day onwards, the mortality rate in both the control group and the group receiving the entire OMP serum stabilized (Figure 2A,B). The immunoprotective efficacy of the entire OMP serum against *P. fluorescens* was 61.5% (*p* < 0.01), while its efficacy against *A. hydrophila* was 57.1% (*p* < 0.01) (Supplementary Table S2). Therefore, the entire OMP serum has immune protective activity against infection by *P. fluorescens* and *A. hydrophila* in fish.

Following passive immunization of *C. auratus* with the entire antiserum of OMP and challenging with *P. fluorescens* or *A. hydrophila*, the antioxidant-related factors (MDA, CAT, GSH-Px, and SOD) decreased (*p* < 0.05) in the *C. auratus* serum (Figure 2C,D) compared to the NC group. Additionally, the mRNA expressions of inflammation factor genes (*il-6*, *il-8*, *tnf-α*, and *il-1β*) significantly decreased (*p* < 0.05) in the kidney, spleen, and gill (Figure 2E,F). These results indicate that the entire OMP antiserum exhibited reductions in inflammatory/oxidative responses associated with protection following bacterial challenge.

Thus, the entire OMP mouse serum had immunoprotective efficacy, and it was necessary to assess which OMP antiserum of *P. fluorescens* had an immune ability.

### 3.2. Cloning, Expression, and Purification of P. fluorescens OMPs

**Figure 3:**
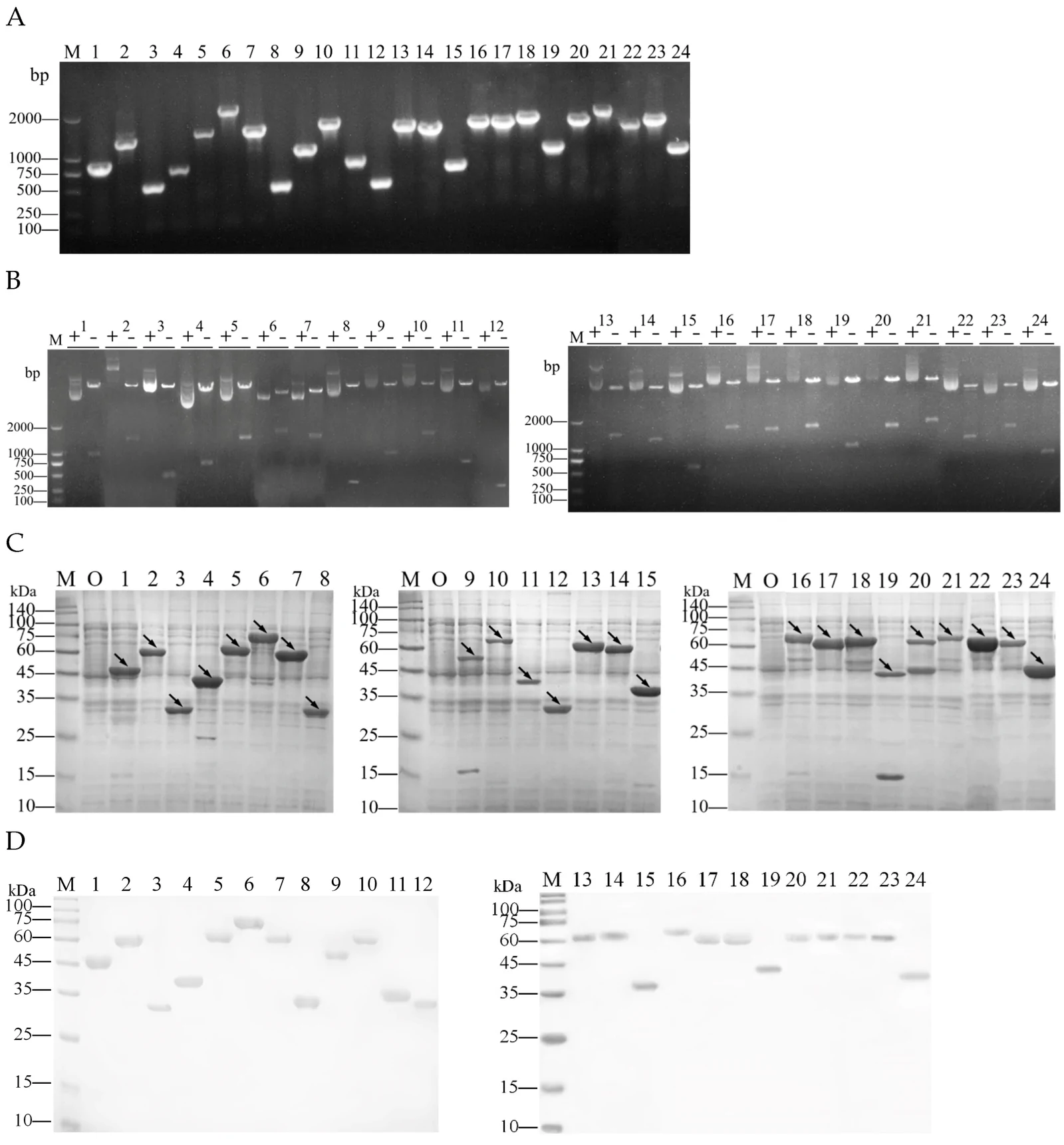
Cloning, expression, and purification of *P. fluorescens* OMPs. (**A**) PCRamplification of the OMP genes. (**B**) Double restriction enzyme of the endonuclease of the recombinant OMP gene plasmid. (**C**) Expression of OMPs. (**D**) Purification of OMPs. (M) Protein marker. (O) Uninduced strain. (+) Recombinant OMPs’ plasmids. () Recombinant OMPs’ plasmids with double enzyme digestion. Numbers 1–24 represent the OMPs of PF0542, PF1068, PF1110, PF1380, SurA, PF1756, PF1798, PF1944, SlyB, PF2253, ExbB, PF2332, PF2361, PF2405, LolA, PF2855, PF2971, PF3127, PF3231, PF3256, PF3269, PF4591, PF4616, and OmpV, respectively.

With *P. fluorescens* genomic DNA as the template, 24 OMP genes were obtained using PCR amplification with the expected size (Figure 3A). The recombinant plasmids of 24 OMP genes were digested with two corresponding endonucleases. Then, the OMP gene band and pET-32a plasmid vector were obtained with the expected size (Figure 3B). The gene sequencing analysis showed that the OMP genes were consistent with the NCBI Gene Bank. After 24 recombinant plasmids were transformed into the *E. coli* BL21 strain, SDS-PAGE protein electrophoresis indicated that the molecular weight of the 24 OMPs was consistent with the expected size (Figure 3C). The 24 purified OMPs had a single band, and the molecular weight of the OMPs was consistent with theory (Figure 3D). Thus, 24 OMPs were cloned, expressed, and purified successfully.

### 3.3. Specificity and Titer of OMP Mouse Antisera

**Figure 4:**
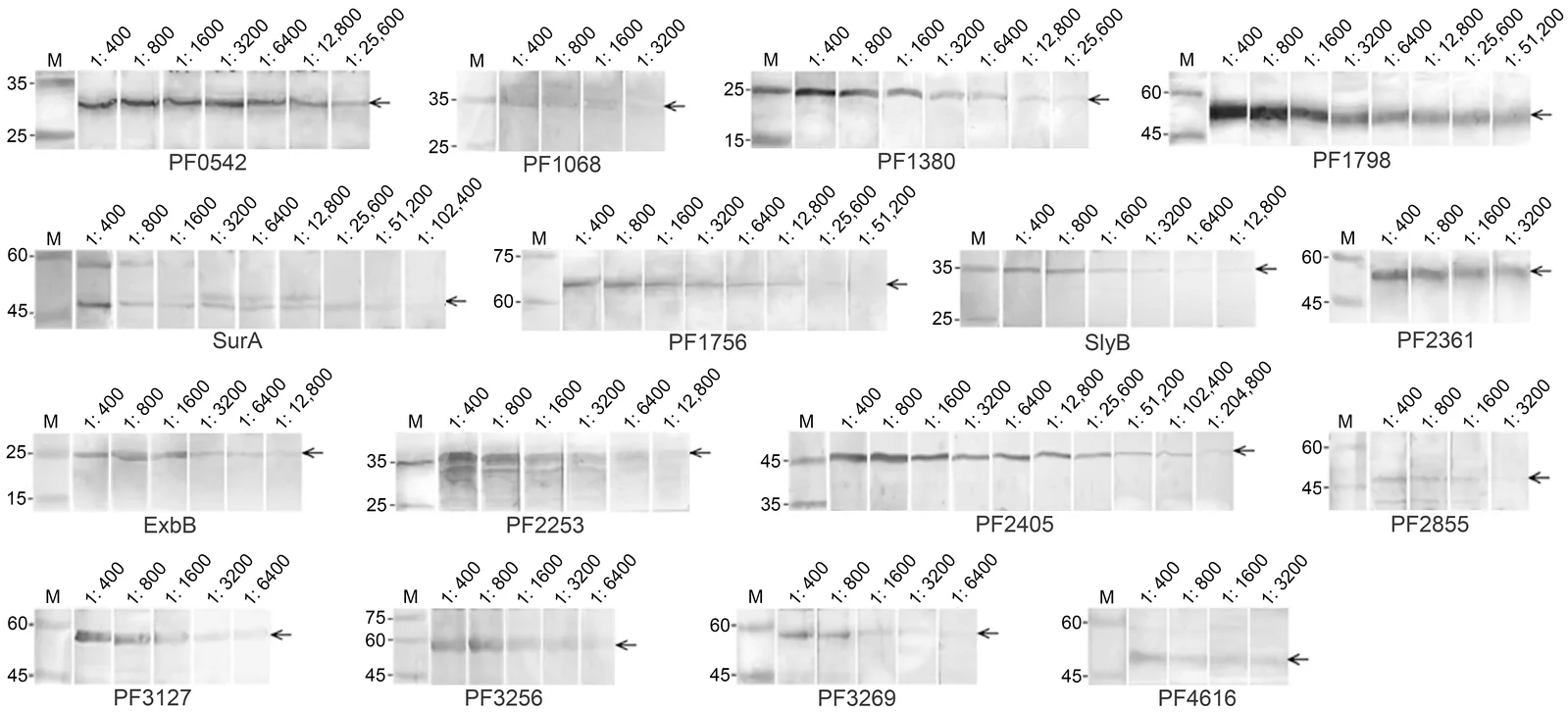
Specificity and titer of anti-OMP mouse sera using Western blotting. (M) Protein marker.

Mice were immunized with 24 OMPs to prepare the antiserum, and the specificity and titer of the serum were evaluated using Western blotting. The results showed that only 16 of the 24 OMP antisera produced specific bands with adequate specificity and titer (Figure 4). The remaining 8 OMPs either failed to produce detectable specific bands or produced non-specific multiple bands in Western blotting, indicating that these proteins did not elicit antibodies capable of recognizing native epitopes. Therefore, only the 16 OMPs with verified specific antisera were proceeded to passive immunoprotection screening, and the titers of the 16 OMP sera are shown in Table 2.

### 3.4. Passive Immunoprotective Rate of the 16 OMP Mouse Sera

After the 16 OMP sera were immunized, *C. auratus* were challenged with *P. fluorescens*. The passive immunoprotective rates of the 16 OMP sera are shown in Table 2. The immune protection rates of the following five OMP sera (*p* < 0.01) were higher than others: PF2253 (78.6%), PF4616 (78.6%), SurA (71.4%), PF1798 (71.4%), and PF0542 (64.3%) (Table 2). Therefore, the passive and active immunoprotective abilities of these five OMPs (PF2253, PF4616, SurA, PF1798, and PF0542) should be evaluated further.

### 3.5. Passive Protective Abilities of the Five Candidate OMP Mouse Sera to P. fluorescens

**Figure 5:**
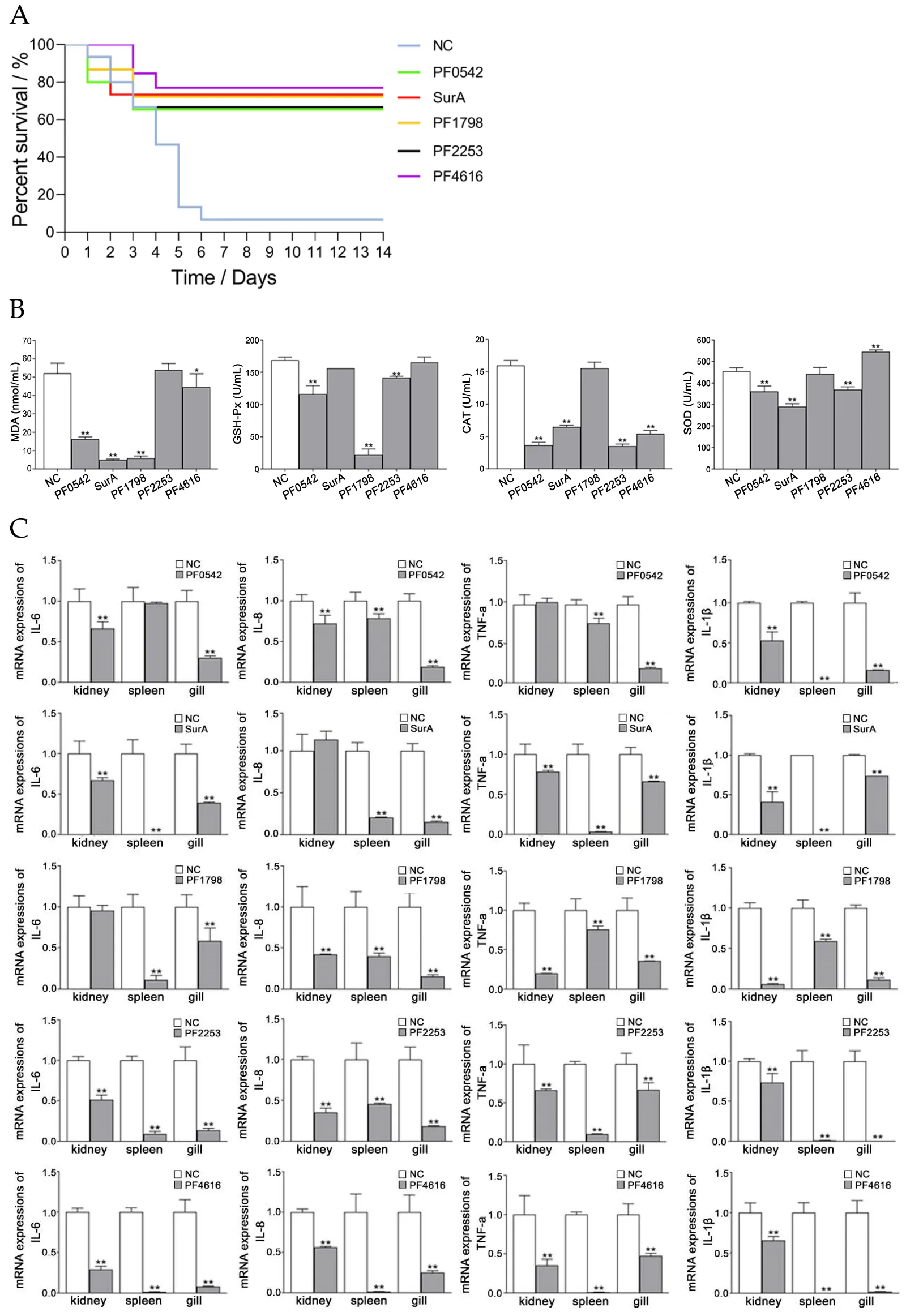
Passive protective abilities after immunizing 5 OMP sera and challenging with *P. fluorescens* in *C. auratus*. * *p* < 0.05, ** *p* < 0.01 (compared with the control). (**A**) Percent survival of *C. auratus* after challenging with *P. fluorescens*. (**B**) The antioxidant-related factors decreased (*p* < 0.05) (n = 3). (**C**) The mRNA expressions of inflammation-related genes decreased (*p* < 0.05) (n = 3).

After the five OMP sera were immunized and challenged with *P. fluorescens* in *C. auratus*, high mortality was observed in the control group within 6 days. On the seventh day, the percentage of survival gradually stabilized in the control group and the five OMP groups (Figure 5A). In the five OMP serum groups, the levels of the four antioxidant-related factors (MDA, CAT, GSH-Px, and SOD) were significantly decreased compared with the control group (*p* < 0.05) in *C. auratus* serum (Figure 5B). Interestingly, the mRNA expressions of inflammation factor genes (*il-6*, *il-8*, *tnf-α*, and *il-1β*) significantly decreased (*p* < 0.05) in the kidney, spleen, and gill (Figure 5C).

**Figure 6:**
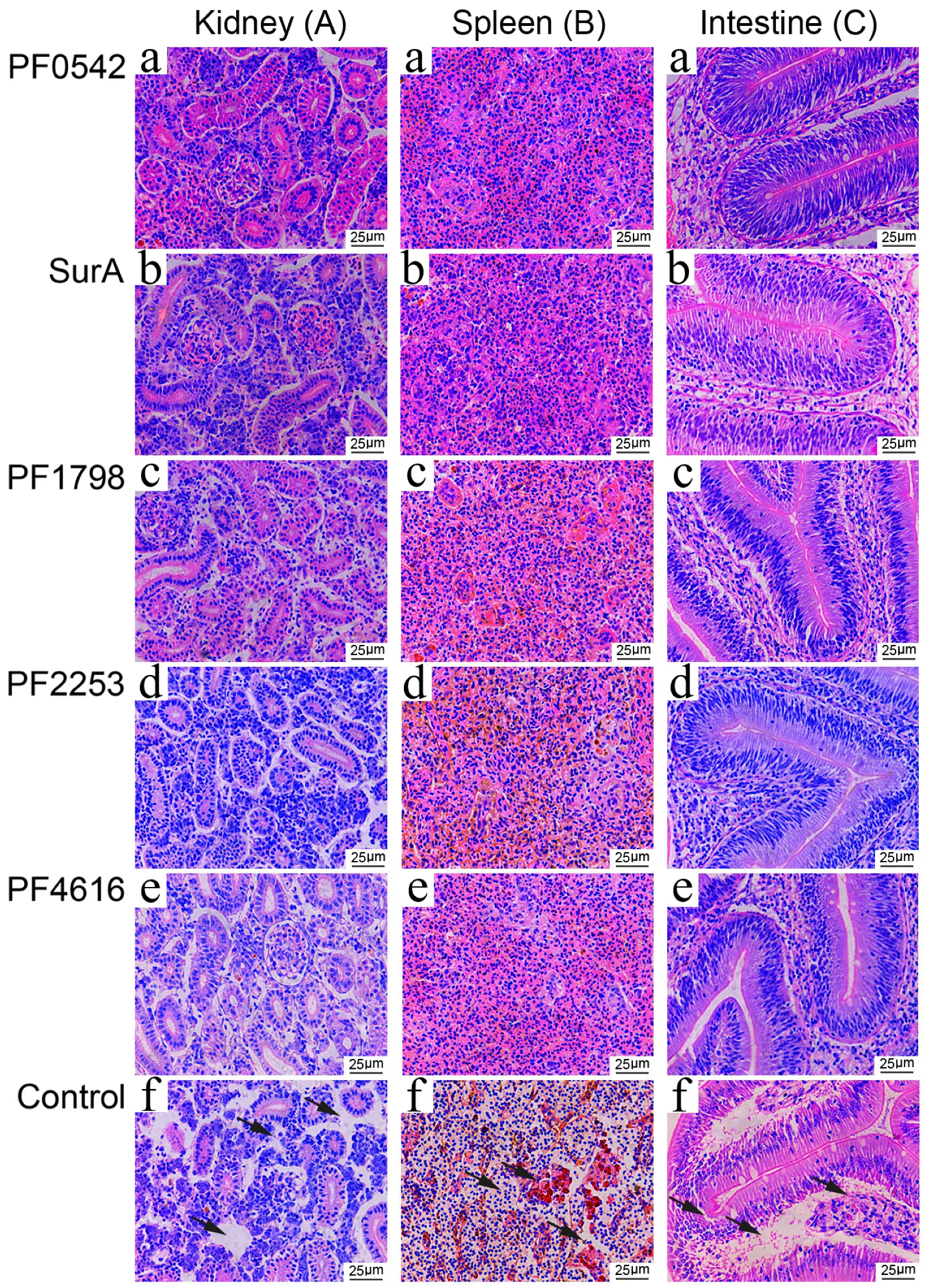
Visceral histopathology after immunizing five OMP sera and challenging with *P. fluorescens* in *C. auratus*. (**A**–**C**) represent the kidney, spleen, and intestine, respectively. (**a**–**f**) represent *C. auratus* immunized with PF0542, SurA, PF1798, PF2253, PF4616, and blank serum, respectively. Unlike in the five OMP sera groups, the kidney structure was incomplete, the glomerular cells degenerated, the intercellular space expanded, and the kidney cells were apoptotic in the control group (**A**); the density of splenic tissue cells decreased, and the structure of the spleen was incomplete and accompanied with hemorrhage in the control group (**B**); and intestinal villus necrosis and shedding, structural disorder, cell degeneration, and apoptosis were observed in the control group (**C**).

**Figure 7:**
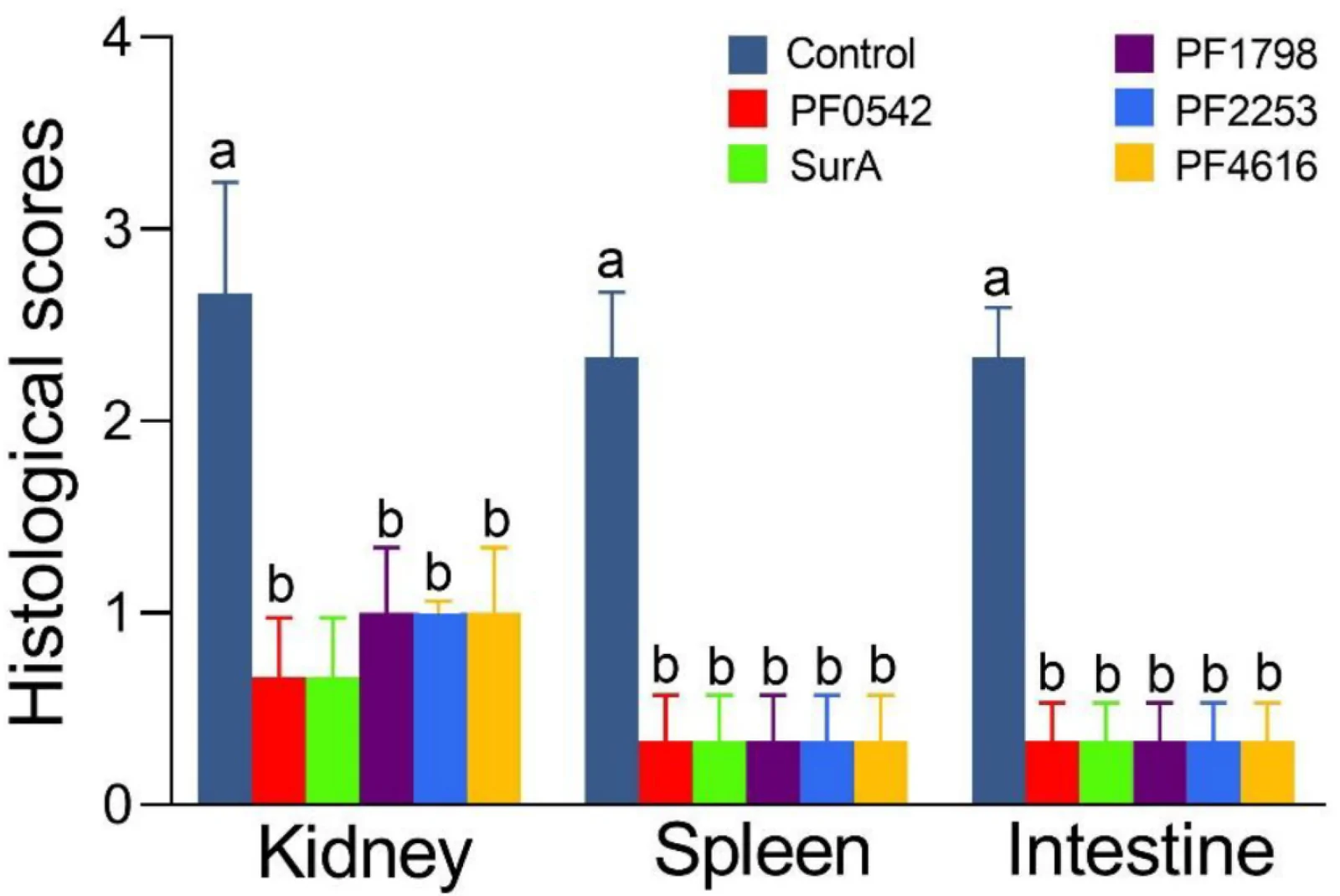
The lesion quantitative scores of kidney, spleen and intestine (n = 3). Statistical comparisons with the control group (NC) reveal significant differences. a, b indicates statistically different groups, and different letters indicate significant differences between the two groups (*p* < 0.05). Among the five OMP sera (PF0542, SurA, PF1798, PF2253, and PF4616), groups denoted by the same letter were not significantly different (*p* > 0.05), whereas the histological scores in the five OMP sera groups were significantly lower than those in the control group, as indicated by different letters (*p* < 0.05).

Tissue pathology sections were prepared following the challenge with *P. fluorescens* in *C. auratus*. In the control, *C. auratus* kidney experienced an incomplete cell structure, with apoptotic glomerular cells and an expanded intercellular septum (Figure 6(Aa–Af)). Similarly, the control group showed a decrease in the number of cells as well as an enlarged gap between splenic cells (Figure 6(Ba–Bf)). Additionally, the control group displayed apoptosis and degeneration of intestinal cells, with an increase in intestinal cell gaps (Figure 6(Ca–Cf)). However, in the five OMP sera groups, there was evidence of a complete structure for the kidney, spleen, and intestine, with neatly arranged and densely packed cells (Figure 6). Moreover, the lesion scoring and quantitative pathology results of the kidneys, spleens, and intestines showed that the scores of the OMPs group were significantly lower than the control group (*p* < 0.05), indicating that the tissue lesion of the OMPs group was lower than the control group (Figure 7).

The findings imply that the five OMP sera (PF0542, SurA, PF1798, PF2253, and PF4616) have an immune protective rate and antioxidant ability, can reduce inflammation, and can maintain the integrity of splanchnic tissue for *C. auratus* against *P. fluorescens*. Therefore, the five OMP sera had passive protective abilities.

### 3.6. Passive Cross-Protective Abilities of the Five Candidate OMP Mouse Sera to A. hydrophila in C. auratus

**Figure 8:**
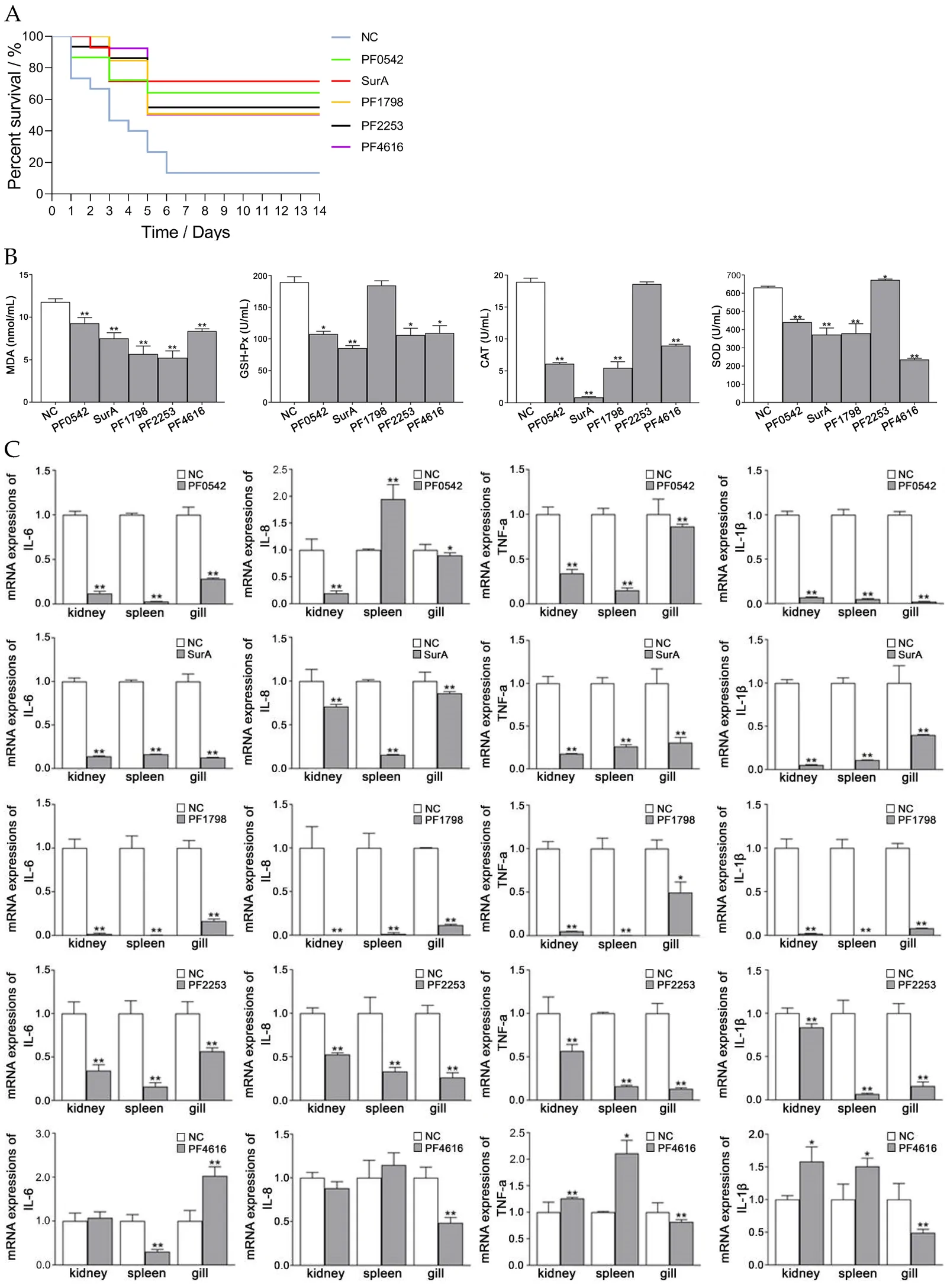
Passive cross-protective abilities after immunizing five OMP sera and challenging with *A. hydrophila* in *C. auratus*. * *p* < 0.05, ** *p* < 0.01 (compared with the control). (**A**) Percent survival of *C. auratus* after challenging with *A. hydrophila*. (**B**) The antioxidant-related factors decreased (*p* < 0.05) in the five OMP sera groups in *C. auratus* serum (n = 3). (**C**) The mRNA expressions of inflammation-related genes decreased (*p* < 0.05) in the OMP sera of PF0542, SurA, PF1798, and PF2253, while that of PF4616 was not obvious (n = 3).

To assess passive cross-protective abilities, *C. auratus* was passively immunized with the five OMP sera and challenged with *A. hydrophila*. The survival rate of the control group decreased significantly within 6 days, followed by gradual stabilization on the seventh day (Figure 8A). The immunoprotective rates (RPS) of the five OMP sera (against *A. hydrophila*) reached significance (*p* < 0.05): PF0542 (61.5%), SurA (69.2%), PF1798 (53.8%), PF2253 (53.8%), and PF4616 (53.8%) (Supplementary Table S3). Therefore, these five OMP sera have immune protective efficacy against *A. hydrophila* infection in fish.

Furthermore, in the five OMP sera groups, the mRNA expression levels of antioxidant related factors (MDA, CAT, GSH-Px, and SOD) were significantly reduced (*p* < 0.05) in *C. auratus* (Figure 8B). In the kidney, spleen, and gill tissues of *C. auratus*, there was a noteworthy decrease (*p* < 0.05) in the mRNA expressions of inflammation factor genes (*il-6*, *il-8*, *tnf-α*, and *il-1β*) in the OMP sera groups of PF0542, SurA, and PF1798, while no significant difference was observed for PF4616 (Figure 8C).

**Figure 9:**
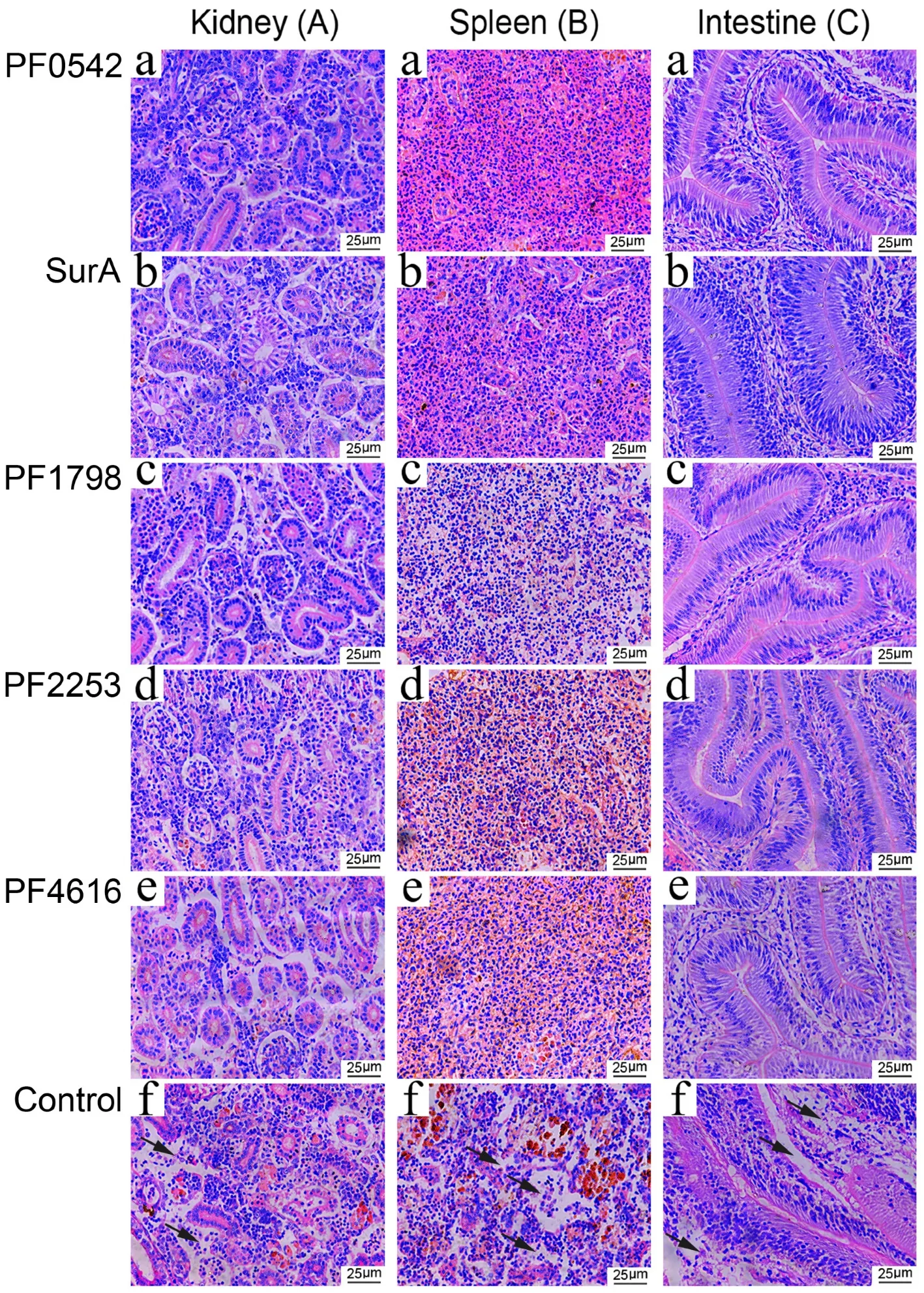
Visceral histopathology after immunizing five OMP sera and challenging with *A. hydrophila* in *C. auratus*. (**A**–**C**) represent the kidney, spleen, and intestine, respectively. (**a**–**f**) represent *C. auratus* immunized with PF0542, SurA, PF1798, PF2253, PF4616, and blank serum, respectively.

**Figure 10:**
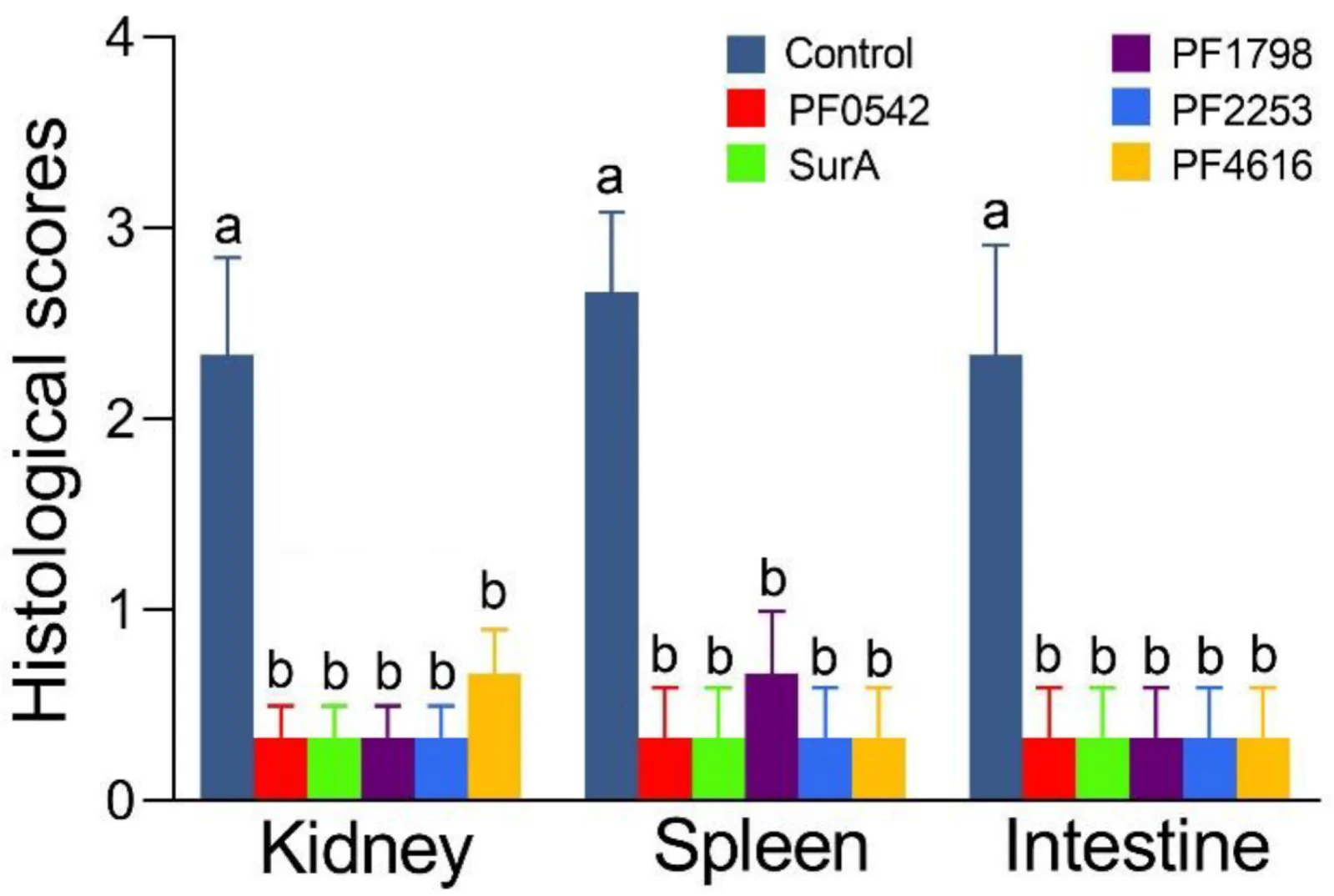
The lesion quantitative scores of kidney, spleen and intestine (n = 3). Statistical comparisons with the control group (NC) reveal significant differences. a, b indicates statistically different groups, and different letters indicate significant differences between the two groups (*p* < 0.05). Among the five OMP sera (PF0542, SurA, PF1798, PF2253, and PF4616), groups denoted by the same letter were not significantly different (*p* > 0.05), whereas the histological scores in the five OMP sera groups were significantly lower than those in the control group, as indicated by different letters (*p* < 0.05).

Tissue pathology sections were prepared following the challenge with *A. hydrophila* in *C. auratus*. In the control group, the *C. auratus* kidney experienced an incomplete cell structure, with apoptotic glomerular cells and expanded intercellular septum (Figure 9(Aa–Af)). Similarly, the control group showed a decrease in the number of cells as well as an enlarged gap between splenic cells (Figure 9(Ba–Bf)). Additionally, the control group displayed apoptosis and degeneration of intestinal cells, with an increase in intestinal cell gaps (Figure 9(Ca–Cf)). However, in the five OMP sera groups, there was evidence of a complete structure for the kidney, spleen, and intestine, with neatly arranged and densely packed cells (Figure 9). Moreover, the lesion scoring and quantitative pathology results of the kidneys, spleens, and intestines showed that the scores of the OMPs group were significantly lower than the control group (*p* < 0.05), indicating that the tissue lesion of the OMPs group was lower than the control group (Figure 10).

These results suggest that the five OMP sera (PF0542, SurA, PF1798, PF2253, and PF4616) have an immune protective rate and antioxidant ability, can eliminate inflammation, and can protect the integrity of visceral tissues and cells to resist *A. hydrophila* infection in *C. auratus*. Therefore, the five OMP mouse sera had passive cross-protective abilities.

Moreover, it is necessary to assess the active protection against both *P. fluorescens* and *A. hydrophila* of the five candidate OMPs (PF0542, SurA, PF1798, PF2253, and PF4616) in *C. auratus*.

### 3.7. Nonspecific Immunity Response of the Five Candidate OMPs in C. auratus

**Figure 11:**
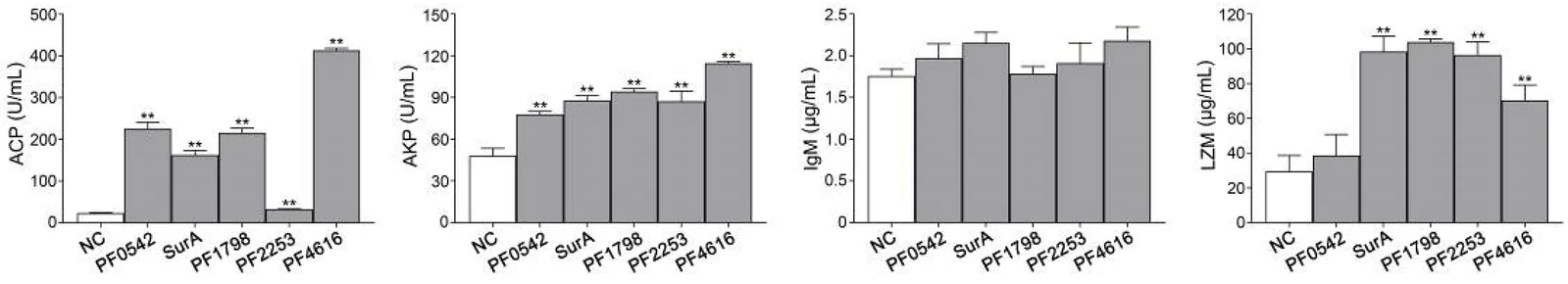
Analysis of immune-related indexes (n = 3). ** *p* < 0.01 (compared with the control).

To identify the nonspecific immunity response of the five OMPs (PF0542, SurA, PF1798, PF2253, and PF4616), the *C. auratus* were immunized with the OMPs, and the immune factors (ACP, AKP, IgM, and LZM) were evaluated in serum. It was shown that the immune factors increased significantly (*p* < 0.05) (Figure 11), suggesting that the five OMPs had the ability to activate a nonspecific immune response in *C. auratus*.

### 3.8. Active Protective Abilities of the Five Candidate OMPs to P. fluorescens in C. auratus

**Figure 12:**
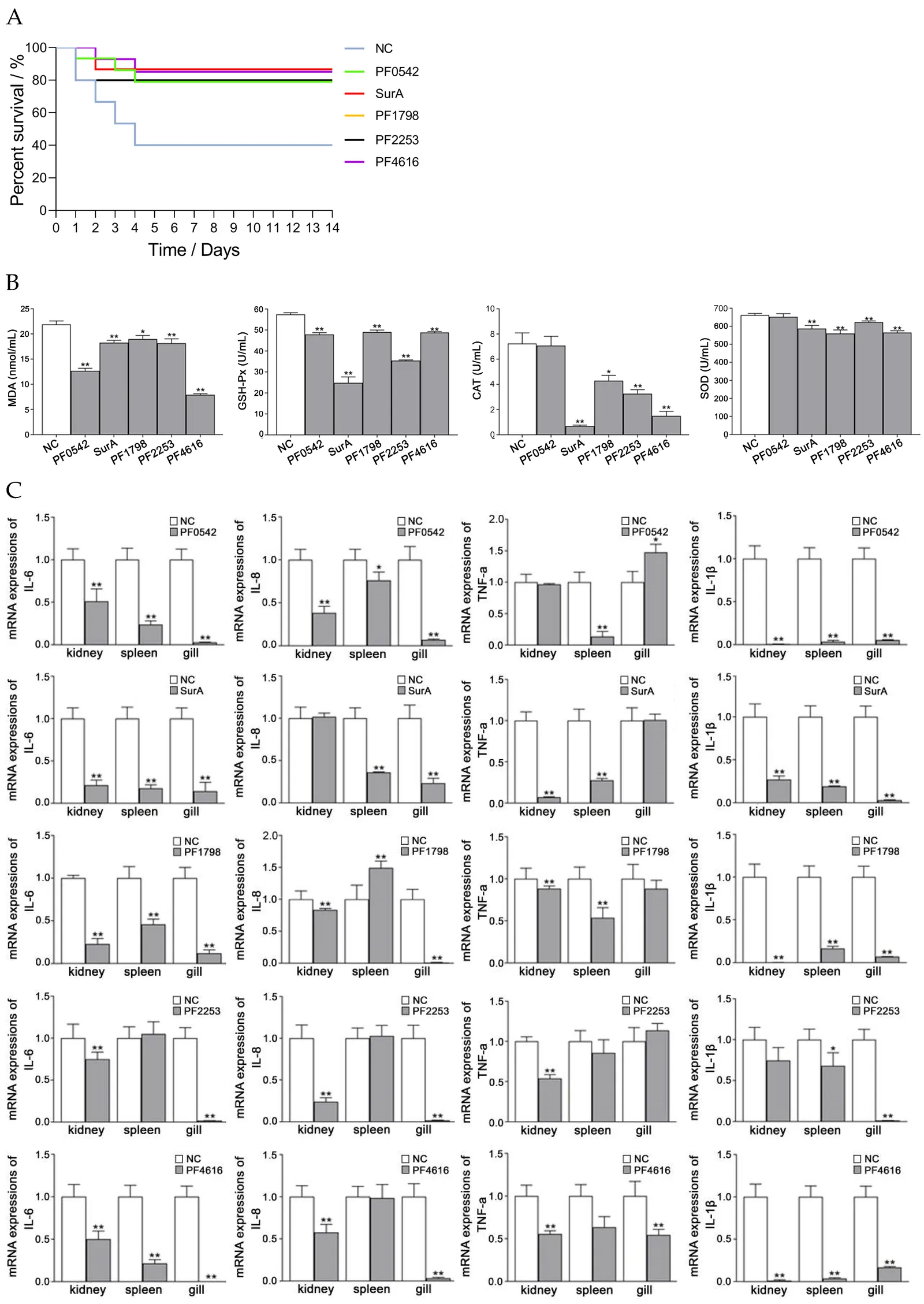
Active protective abilities after immunizing five OMPs and challenging with *P. fluorescens* in *C. auratus*. * *p* < 0.05, ** *p* < 0.01 (compared with the control). (**A**) Percent survival of *C. auratus* after challenge with *P. fluorescens*. (**B**) The antioxidant-related factors decreased (*p* < 0.05) (n = 3). (**C**) The mRNA expressions of inflammation-related genes decreased (*p* < 0.05) (n = 3).

To evaluate the active protective rate, *C. auratus* were immunized with five OMPs and challenged with *P. fluorescens*. After the attack, the *C. auratus* swam slowly and ate less, and high mortality was observed in the control group within 4 days. The mortality of the five OMPs and the control group gradually stabilized after 5 days (Figure 12A). The immunoprotective rates of the five OMPs reached significance (*p* < 0.05): PF0542 (66.7%), SurA (77.8%), PF1798 (77.8%), PF2253 (66.7%), and PF4616 (77.8%) (Supplementary Table S4). Therefore, these five OMPs have immune protective efficacy against *P. fluorescens* infection in fish.

As shown in Figure 12B, the antioxidant-related factors (MDA, CAT, GSH-Px, and SOD) significantly decreased (*p* < 0.05) in the five OMP groups of *C. auratus* serum. As shown in Figure 12C, the mRNA expressions of inflammation factor genes (*il-6*, *il-8*, *tnf-α*, and *il-1β*) significantly reduced (*p* < 0.05) in the kidney, spleen, and gill tissues of the five OMP groups.

**Figure 13:**
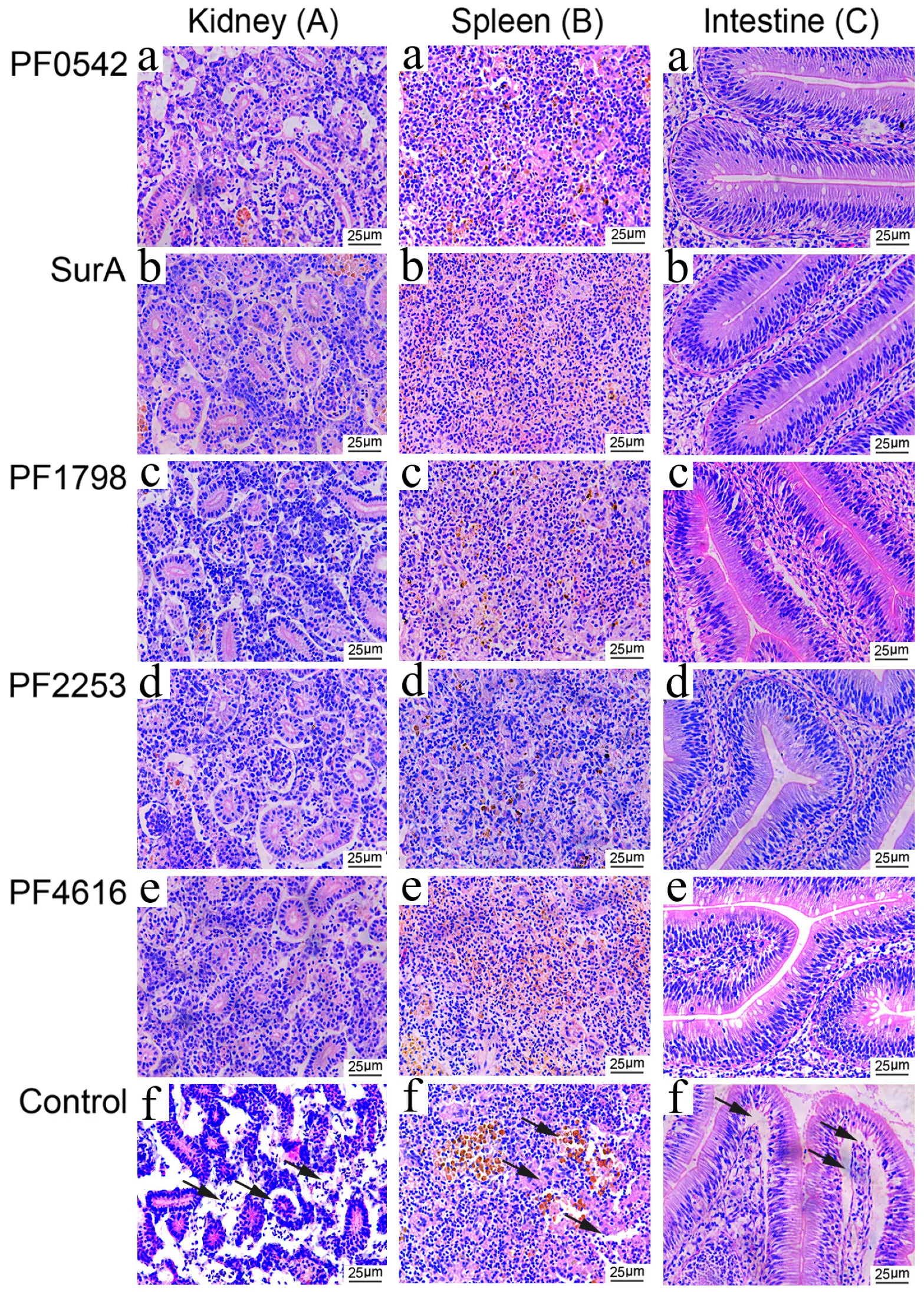
Visceral histopathology after immunizing five OMPs and challenging with *P. fluorescens* in *C. auratus*. (**A**–**C**) represent the kidney, spleen, and intestine, respectively. (**a**–**f**) represent *C. auratus* immunized with PF0542, SurA, PF1798, PF2253, PF4616, and PBS solution, respectively.

**Figure 14:**
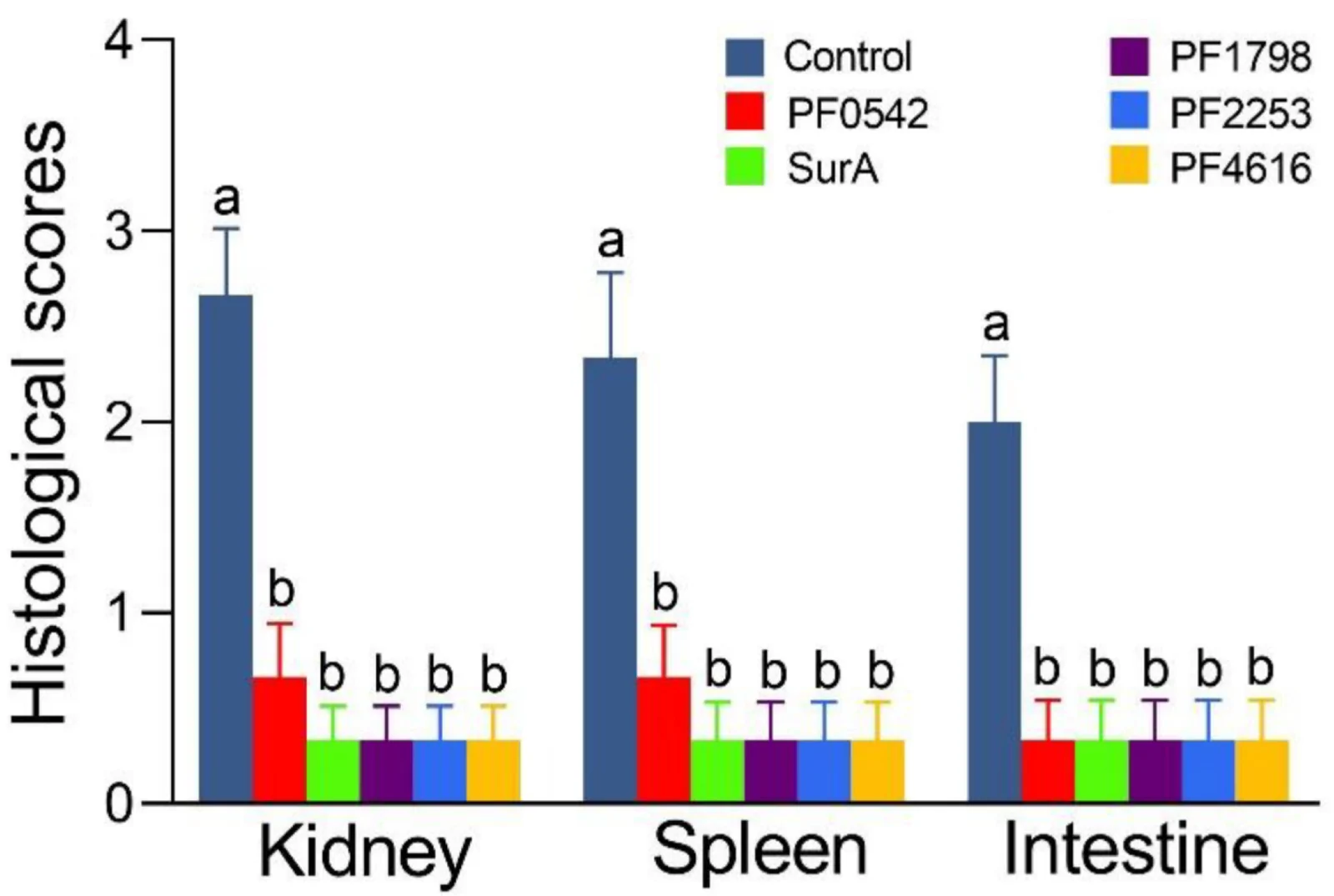
The lesion quantitative scores of kidney, spleen and intestine (n = 3). Statistical comparisons with the control group (NC) reveal significant differences. a, b indicates statistically different groups, and different letters indicate significant differences between the two groups (*p* < 0.05). Among the five OMPs (PF0542, SurA, PF1798, PF2253, and PF4616), groups denoted by the same letter were not significantly different (*p* > 0.05), whereas the histological scores in the five OMPs groups were significantly lower than those in the control group, as indicated by different letters (*p* < 0.05).

Moreover, tissue pathology sections were prepared following the challenge with *P. fluorescens* in *C. auratus*. In the control group, the *C. auratus* kidney experienced an incomplete cell structure, with apoptotic glomerular cells and expanded intercellular septum (Figure 13(Aa–Af)). Furthermore, the control group showed a decrease in the number of cells as well as an enlarged gap between splenic cells (Figure 13(Ba–Bf)). In addition, the control group displayed apoptosis and degeneration of intestinal cells, with an increase in intestinal cell gaps (Figure 13(Ca–Cf)). However, in the five OMP groups, there was evidence of a complete structure for the kidney, spleen, and intestine, with neatly arranged and densely packed cells (Figure 13). Further, the lesion scoring and quantitative pathology results of the kidneys, spleens, and intestines showed that the scores of the OMPs group were significantly lower than the control group (*p* < 0.05), indicating that the tissue lesion of the OMPs group was lower than the control group (Figure 14).

The findings indicate that the five OMPs (PF0542, SurA, PF1798, PF2253, and PF4616) had an immune protective rate, exhibited reductions in inflammatory/oxidative responses associated with protection following bacterial challenge, and sustained the structural integrity of internal organs when confronted with *P. fluorescens* in *C. auratus*. Thus, the five OMPs exhibited active protective functions.

### 3.9. Active Cross-Protective Abilities of the Five Candidate OMPs to A. hydrophila in C. auratus

**Figure 15:**
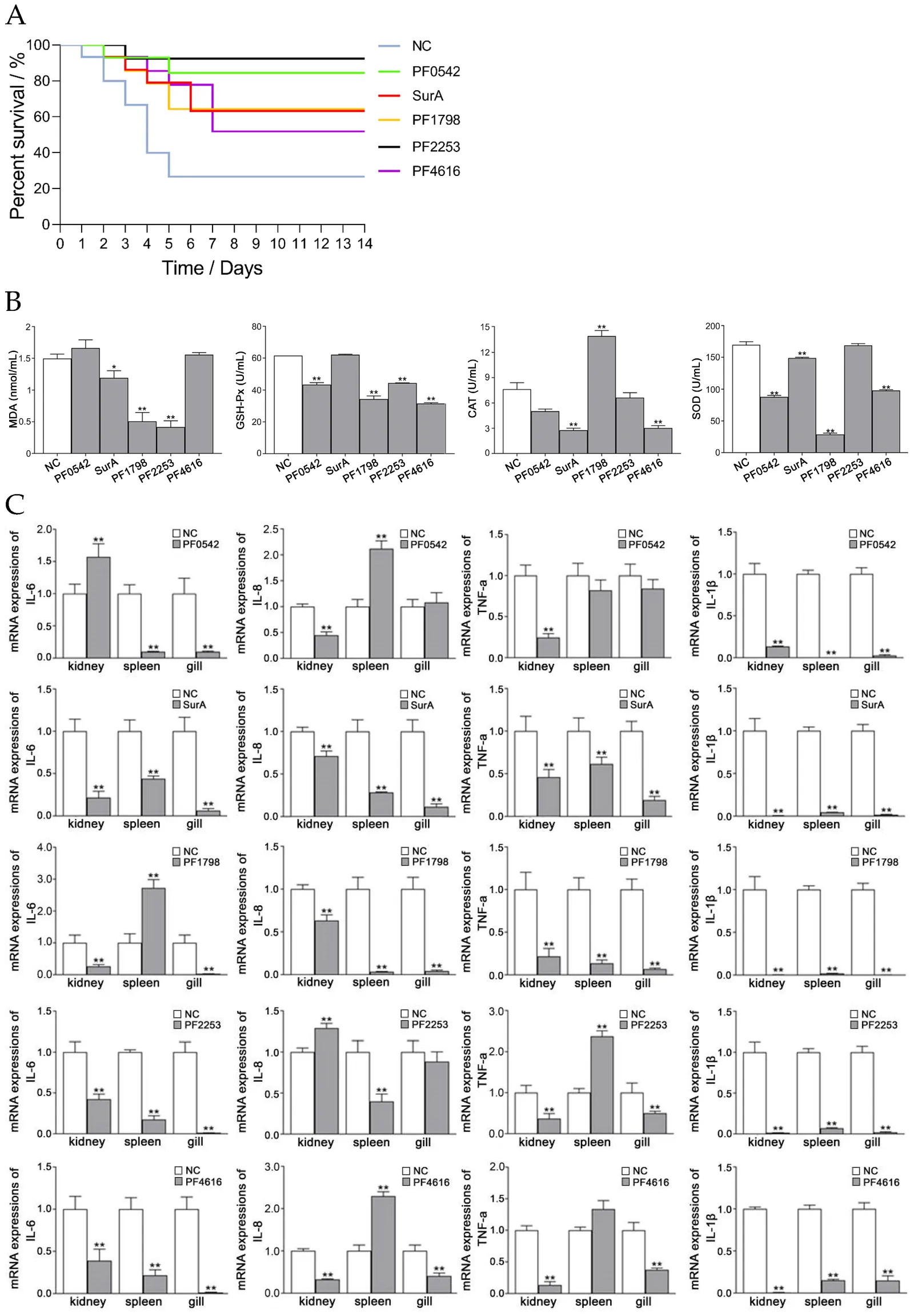
Active cross-protective abilities after immunizing five OMPs and challenging with *A. hydrophila* in *C. auratus*. * *p* < 0.05, ** *p* < 0.01 (compared with the control). (**A**) Percent survival of *C. auratus* after challenge with *A. hydrophila*. (**B**) The antioxidant-related factors decreased (*p* < 0.05) (n = 3). (**C**) The mRNA expressions of the inflammation-related genes decreased (*p* < 0.05) (n = 3).

*C. auratus* were immunized with the five OMPs and challenged to *A. hydrophila* to assess their active cross-protective abilities. In the control group, high mortality of *C. auratus* was observed within 5 days, and the survival rates of the OMP and control groups gradually stabilized after 7 days (Figure 15A). The immunoprotective rates of the OMPs were 81.8% for PF0542, 54.4% for SurA, 54.4% for PF1798, and 90.9% for PF2253 against *A. hydrophila* (*p* < 0.05). However, the immunoprotective rate of PF4616 was 45.5% (*p* > 0.05) (Supplementary Table S5). Therefore, these four OMPs (PF0542, SurA, PF1798 and PF2253) have immune protective efficacy against *A. hydrophila* infection in fish.

In the five OMP groups, the antioxidant-related factors (MDA, CAT, GSH-Px, and SOD) decreased significantly (*p* < 0.05) in *C. auratus* serum (Figure 15B), and the mRNA expressions of the inflammation factor genes (*il-6*, *il-8*, *tnf-α*, and *il-1β*) decreased (*p* < 0.05) in the kidney, spleen, and gill of *C. auratus* compared to control group (Figure 15C).

**Figure 16:**
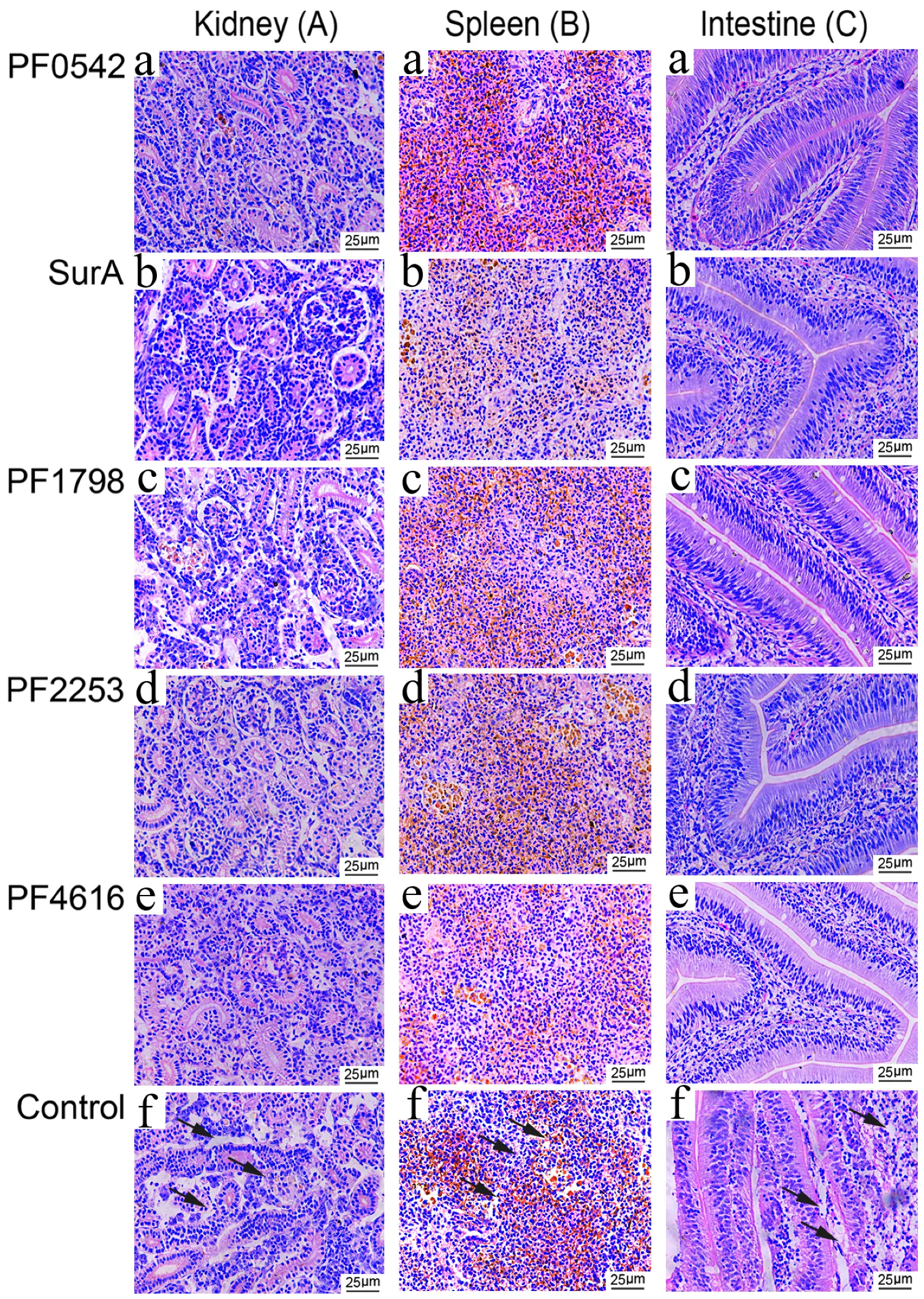
Visceral histopathology after immunizing five OMPs and challenging with *A. hydrophila* in *C. auratus*. (**A**–**C**) represent the kidney, spleen, and intestine, respectively. (**a**–**f**) represent *C. auratus* immunized with PF0542, SurA, PF1798, PF2253, PF4616, and PBS solution, respectively.

**Figure 17:**
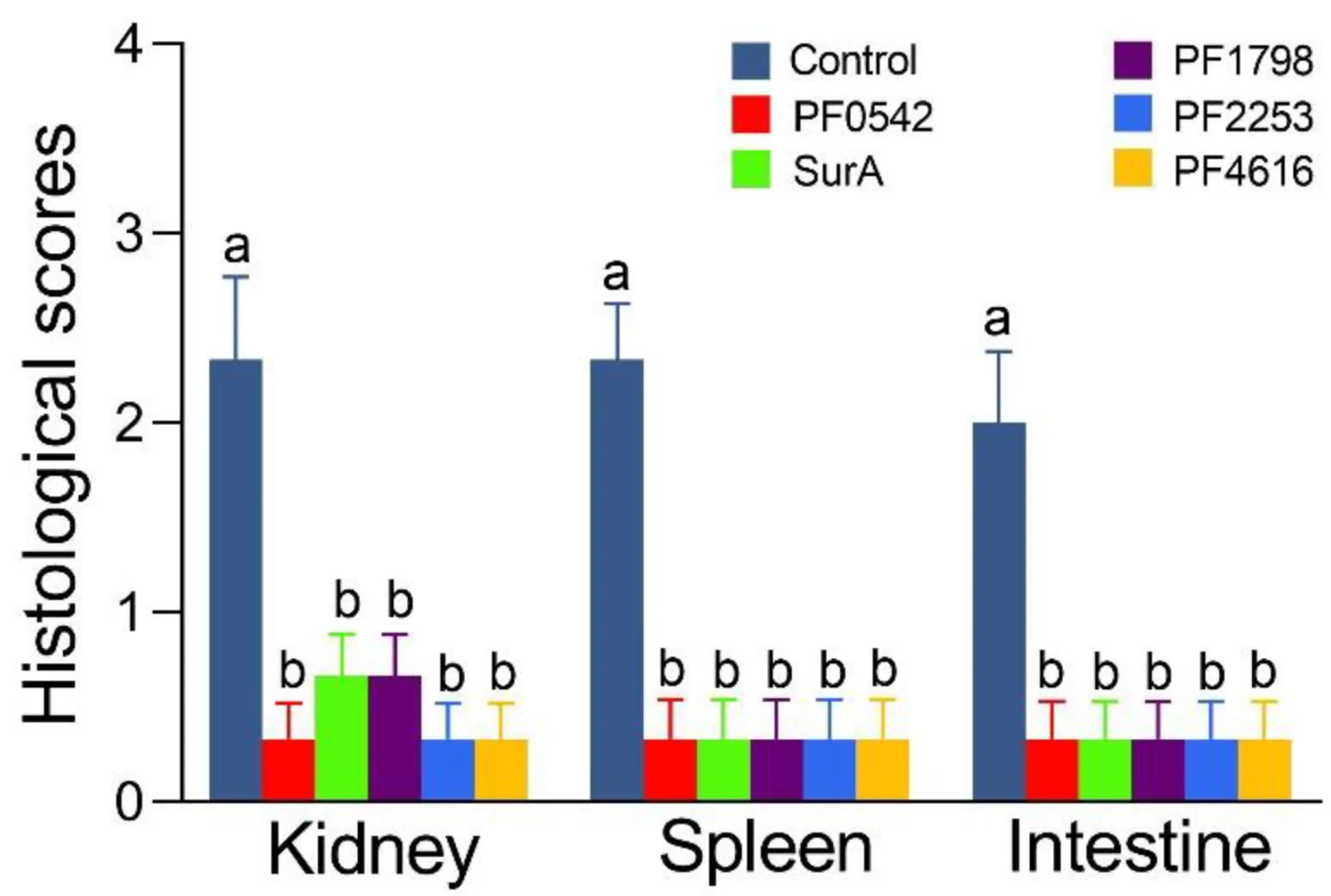
The lesion quantitative scores of kidney, spleen and intestine (n = 3). Statistical comparisons with the control group (NC) reveal significant differences. a, b indicates statistically different groups, and different letters indicate significant differences between the two groups (*p* < 0.05). Among the five OMPs (PF0542, SurA, PF1798, PF2253, and PF4616), groups denoted by the same letter were not significantly different (*p* > 0.05), whereas the histological scores in the five OMPs groups were significantly lower than those in the control group, as indicated by different letters (*p* < 0.05).

Unlike in the five OMP groups, the histopathological sections indicated that the *C. auratus* kidney experienced an incomplete cell structure, with apoptotic glomerular cells and expanded intercellular septum in the control group (Figure 16(Aa–Af)). The control group showed a decrease in the number of spleen cells as well as an enlarged gap between spleen cells (Figure 16(Ba–Bf)). In addition, the control group displayed apoptosis and degeneration of intestinal cells, with an increase in intestinal cell gaps (Figure 16(Ca–Cf)). In the five OMP groups, there was complete tissue structure for the kidney, spleen, and intestine, with neatly arranged and densely packed cells (Figure 16). Moreover, the lesion scoring and quantitative pathology results of the kidneys, spleens, and intestines showed that the scores of the OMPs group were significantly lower than the control group (*p* < 0.05), indicating that the tissue lesion of the OMPs group was lower than the control group (Figure 17).

These results suggest that four OMPs (PF0542, SurA, PF1798, and PF2253) have an immune protective rate and antioxidant ability, eliminate inflammation, and can protect the integrity of visceral tissues and cells to resist *A. hydrophila* in *C. auratus*. Therefore, these four OMPs have active cross-protective abilities.

## 4. Discussion

Vaccines can resist bacterial diseases and have received widespread attention in aquaculture vaccine research [37]. Previous studies have indicated that the OMPs of bacteria could activate animal immune responses and have the potential to be used as polyvalent vaccines [38,39]. Identifying and obtaining OMPs with a good immune protection effect is important [40,41]. In an immune function evaluation, Peng et al. raised antibodies against OMPs of *Vibrio parahaemolyticus* by immunizing mice, and subsequently identified four protective antigens (VP2309, VP0887, VPA0548, and VP1019) through passive immunization and pathogen challenge experiments in fish [42]. Using a comparable approach, Liu et al. obtained 15 OMPs of *A. hydrophila* and prepared corresponding mouse antiserum, and identified four protective antigens (OmpW, OmpAII, P5, and AHA2685) [43]. Addition, mice were used as the mammalian immunization model for initial polyclonal antiserum generation because: (1) the mice are the standard model for rapid polyclonal antibody production, providing sufficient serum volume for titer determination and passive challenge experiments; (2) mouse antisera are well-characterized for Western blotting analysis with commercially available secondary antibodies [38,42,43]. In this study, 24 OMPs of *P. fluorescens* were obtained using a molecular cloning method, and 16 OMP-specific antibodies with high titers were prepared by immunizing mice, giving rise to the screening and identification of cross-protective immunogens of *P. fluorescens* OMPs.

Passive immunity refers to the specific immunity effects obtained by animals directly from immunizing antibodies. It has a quick effect and does not need to pass an incubation period. Once immunized, animals can obtain immunity [44,45]. The evaluation of passive immune protection is an effective way to screen protective immunogens and is useful for the development of passive immunity vaccines [46,47]. Peng et al. immunized zebrafish and challenged pathogenic bacteria with the mouse antiserum of 17 OMPs of *V. parahaemolyticus*. A passive immune protection test showed that the OMP antisera of VP0887, VP1019, VPA0548, and VP2309 could resist *V. parahaemolyticus*, *P. fluorescens*, and *A. hydrophila* [41]. Li et al. (2010) showed that the OMP mouse antiserum of VPA1435, VP0764, VPA1186, VP1061, and VP2850 of *V. parahaemolyticus* had an immune ability against *V. parahaemolyticus* infection and that VP1061 and VP2850 had a passive cross-protective effect against *P. fluorescens*, *A. hydrophila*, and *V. alginolyticus* infection [48]. In the current study, after the entire OMP serum had been immunized to *C. auratus* prior to infection with pathogenic bacteria, the antioxidant factors and the mRNA levels of the inflammation genes significantly decreased (*p* < 0.05), and they held a significant protection rate to resist *P. fluorescens* and *A. hydrophila* infection. Thus, the entire OMP antiserum has passive protection against both *P. fluorescens* and *A. hydrophila*. Furthermore, we immunized *C. auratus* with 16 OMPs of the antiserum of *P. fluorescens* and challenged pathogenic bacteria. The results showed that OMP sera of PF0542, SurA, PF1798, PF2253, and PF4616 reduced the expressions of the antioxidant factors and the inflammation genes, reduced histopathological damage, and had an immune protective rate (*p* < 0.05) against *P. fluorescens* and *A. hydrophila* infection. Antioxidant factors reflect the activity of antioxidation in animals. A decreased expression level indicates that the degree of antioxidation damage is low, which is an indicator of drug function evaluation [49]. In this context, the higher levels of MDA, CAT, SOD, and GSH-Px in the NC group reflect reactive upregulation in response to severe bacterial-induced oxidative burden, while their relative decrease in OMP-immunized groups—accompanied significantly reduced pro-inflammatory cytokines indicates that OMP-immunized fish experienced lower oxidative burden due to better pathogen control. The expressions of inflammation-related genes reflect the level of inflammation in animals and can be used to identify the anti-inflammatory effects of drugs [50]. The integrity of the histopathological structure can be used to evaluate the protective effect of drugs on tissues and organs [51]. The calculation of the immune protection rate directly reflects the mortality of animals, which is an important indicator for evaluating a drug’s immune-protective ability [52]. Therefore, the OMP antiserum of PF0542, SurA, PF1798, PF2253, and PF4616 has passive protection against both *P. fluorescens* and *A. hydrophila* infections in *C. auratus*. Moreover, *P. fluorescens* and *A. hydrophila* are important pathogens in freshwater aquaculture [6,53]. These results suggest that PF0542, SurA, PF1798, PF2253, and PF4616 hold potential value as protective immunogens for passive immune candidate vaccines in freshwater aquaculture. Further, the persistence and functional duration of mouse immunoglobulins in fish were not experimentally determined, and the passive protection data primarily reflect antibody activity in the acute phase of infection. Future studies could assess the pharmacokinetics of passively transferred antibodies in fish, including serum half-life and tissue distribution, to better characterize the model. Additionally, in this study, the passive-immunization experiment is a screening model for protective antibody activity and does not directly represent the endogenous immune response generated by vaccination in fish.

Active immune protective evaluation aims to immunize animals with proteins to produce humoral and cellular immunity and to activate the specific immunity of the animals [54,55]. It assesses the immunoprotection of proteins by challenging pathogenic bacteria and establishing the development of active immune vaccines [56,57]. Peng et al. actively immunized zebrafish with *V. parahaemolyticus* OMPs of VP2309, VP0887, VPA0548, and VP1019 identified from passive immune evaluation and attacked pathogenic bacteria. They found that these four OMPs had a protective rate against *V. parahaemolyticus*, *A. hydrophila*, and *P. fluorescens*, which are polyvalent protective immunogens [42]. In a previous study, we obtained the *A. hydrophila* OmpW protein through passive immune screening, actively immunized it to *C. auratus*, and attacked pathogenic bacteria. The OmpW protein was found to have immunoprotective abilities against *A. hydrophila* and *V. alginolyticus*, which are polyvalent protective immunogens [43]. In this research, five OMPs of PF0542, SurA, PF1798, PF2253, and PF4616 identified by passive immune screening were actively immunized in *C. auratus*, and these five OMPs could activate the innate immunity response of *C. auratus*. Moreover, the results of attacking pathogenic bacteria showed that the five OMPs had an immune protective rate and could reduce the expressions of antioxidant-related factors and inflammation-related genes and the tissue pathological damage caused by *P. fluorescens* and *A. hydrophila* infections. Therefore, PF0542, SurA, PF1798, PF2253, and PF4616 have active protection against both *P. fluorescens* and *A. hydrophila* and hold potential value as protective immunogens for active immune candidate vaccines in freshwater aquaculture. In this study, Freund’s adjuvant and intraperitoneal injection are laboratory-based immunization strategies and are not directly applicable to large-scale aquaculture vaccination. Freund’s adjuvant should be replaced with commercially approved fish adjuvants such as Montanide ISA 763A, which has been validated in multiple fish species and provides potent immune responses with fewer adverse reactions [58,59]. While intraperitoneal injection is effective, alternative delivery routes such as immersion and oral vaccination [60] should be explored in future work. Further, antigen encapsulation using nanomaterials (e.g., chitosan nanoparticles, PLGA microcapsules) or spore-based delivery systems may protect recombinant protein antigens from degradation and enhance mucosal immune responses.

In this study, we screened 24 *P. fluorescens* OMPs—the largest such effort to date—and identified five candidates (PF0542, SurA, PF1798, PF2253, and PF4616) with cross-protective efficacy against both *P. fluorescens* and *A. hydrophila*, confirmed by passive and active immunization. Four OMPs (PF0542, PF1798, PF2253, and PF4616) are novel protective antigens in fish, and SurA is validated as a vaccine candidate for the first time. Comprehensive evaluation of immune, antioxidant, inflammatory, and histopathological responses further elucidated their protective mechanisms. To elucidate the underlying mechanism of the five cross-protective antigens (PF0542, SurA, PF1798, PF2253, and PF4616), we performed NCBI BLAST database (https://blast.ncbi.nlm.nih.gov/Blast.cgi, accessed on 28 August 2026) searches against the *A. hydrophila* proteome using the sequences of PF0542, SurA, and PF2253 of *P. fluorescens* based on available data in the NCBI database. The Query cover values indicated that PF0542 shared 54% and 43% coverage with YfiB and an OmpA family protein of *A. hydrophila*, respectively; SurA shared 74% and 22% coverage with SurA and peptidylprolyl isomerase of *A. hydrophila*, respectively; and PF2253 shared 95%, 79%, and 60% coverage with an efflux transporter outer membrane subunit, OprM, and a TolC family protein, respectively (Supplementary Table S6). These results suggest that the sequence similarities may lead to the generation of cross-reactive polyclonal antibodies during antigen presentation, which bind to corresponding proteins of *A. hydrophila*, forming antigen–antibody complexes and facilitating phagocyte-mediated bacteria clearance [42,43]. However, the BLAST-based sequence similarity indicates potential homology but does not confirm shared epitopes or cross-reactive antibody recognition, and that experimental validation (e.g., antibody cross-reactivity assays, epitope mapping) is required to definitively establish the mechanism. Collectively, these findings provide preliminary support for the hypothesis that PF0542, SurA, and PF2253 confer cross-protective immunity against *A. hydrophila* infection. Furthermore, the upregulation of ACP, AKP, and LZM indicates non-specific immune activation in this study, whereas the specificity and functionality of the antibody response warrant further investigation. Moreover, this study measured total serum IgM levels as an indicator of overall humoral immune activation in fish, and it did not perform antigen-specific ELISA to quantify antibodies directed against each individual OMPs. Future studies will incorporate antigen-specific ELISA to monitor antibody titers of OMPs and their correlation with protective efficacy, which would provide a more complete understanding of the immune mechanisms involved.

It is necessary to explicitly distinguish between (a) direct anti-inflammatory/antioxidant activities of the OMPs themselves and (b) indirect effects mediated through reduced bacterial burden following successful vaccination. This study clearly indicates that the more parsimonious interpretation of the data is the latter—that the observed reduction in inflammatory cytokines and oxidative markers reflects lower bacterial burden in OMP-immunized fish, which is supported by the increased survival rates, preserved tissue architecture, and enhanced immune parameters in the OMP groups. However, the experimental design does not distinguish between direct and indirect anti-inflammatory activities of the OMPs, and future experiments should include direct bactericidal assays or macrophage stimulation assays with OMPs. Additionally, future studies are needed to generate long-term protection data, antibody characterization, and mechanistic insights into cross-protection. Moreover, further studies against additional aquaculture pathogens are needed to fully establish the polyvalent potential of these OMPs candidates.

## 5. Conclusions

In summary, 24 OMPs of *P. fluorescens* were cloned and purified, and the corresponding 16 OMP mouse sera were prepared. Passive and active immunization showed that five OMPs (PF0542, SurA, PF1798, PF2253, and PF4616) could activate innate immune activity, have an immune protection rate, downregulate the expressions of antioxidant-related factors (MDA, SOD, CAT, and GSH-Px) and inflammation genes (*il-6*, *il-8*, *il-1β*, and *tnf-α*) associated with protection following bacterial challenge, and have protective abilities for visceral organs after being infected with *P. fluorescens* or *A. hydrophila* in *C. auratus*. Four OMPs (PF0542, PF1798, PF2253, and PF4616) are newly identified protective antigens in fish, and SurA is reported as a vaccine candidate for the first time. This study contributes by identifying five OMPs (PF0542, SurA, PF1798, PF2253, and PF4616) that could be vaccine candidates for passive and active immunization in aquaculture.

## Figures and Tables

**Table 1:** Primer information of *P. fluorescens* genes.

Genes	GenBank No.	MW (bp)	MW (kDa)	Res. Site	Primer Sequence (5′-3′)
*PF0542*	SAMN04488487-0542	816	30	*Bam*H Ι *Xoh* Ι	F: ACCGGATCCATGAGCCCGATGATCCGT
					R: CCACTCGAGTCAGCGCGCAGCGCCTAA
*PF1068*	SAMN04488487-1068	1224	45	*Bam*H Ι *Xoh* Ι	F: CAAGGATCCATGCGCGAGTTATTCACG
					R: CCGCTCGAGCTACCAAAGGGCCAGGTC
*PF1110*	SAMN04488487-1110	492	18	*Bam*H Ι *Xoh* Ι	F: ACTGGATCCTTAGTCCGCGATCACCAC
					R: CACCTCGAGTTGTTCTCGAATGCGCGA
*PF1380*	SAMN04488487-1380	699	26	*Bam*H Ι *Xoh* Ι	F: AGTGGATCCATGAACAAGTCCCTGCTC
					R: GGTCTCGAGCTAGAACTTGTAGCCGAT
*SurA*	SAMN04488487-1499	1341	49	*Bam*H Ι *Xoh* Ι	F: AGGGGATCCGTGAACGTGAAGACCAAG
					R: GTGCTCGAGTCACTTCGGTGCCTGGTC
*PF1756*	SAMN04488487-1756	1947	71	*Bam*H Ι *Xoh* Ι	F: GTGGGATCCGTGATAAAGCCTCTTGCC
					R: CTCCTCGAGTCAAAACATCCATTGCACG
*PF1798*	SAMN04488487-1798	1365	50	*Bam*H Ι *Xoh* Ι	F: ACCGGATCCATGAAGAAGTCAACATTG
					R:ACCCTCGAGCTAGAACAGTGGCAGCGAG
*PF1944*	SAMN04488487-1944	426	16	*Bam*H Ι *Xoh* Ι	F: GCAGGATCCATGGGCCTGCATTTGAAT
					R: GAGCTCGAGTCATTTCTTGGCTGCCGTC
*SlyB*	SAMN04488487-1950	927	34	*Bam*H Ι *Xoh* Ι	F: ACCGGATCCATGCTTTTTTCCCGTAAG
					R: CCACTCGAGTTAAACGTTAGGGCAAGG
*PF2253*	SAMN04488487-2253	1452	53	BamH Ι Xoh Ι	F: AGGGGATCCATGAAGTCATTTGTCTGGC
					R: GGACTCGAGTCAGCCTGCAGGAAGGGA
*ExbB*	SAMN04488487-2331	720	26	*Bam*H Ι *Xoh* Ι	F: ACCGGATCCATGGCATTAGCATCTCCA
					R: GCTCTCGAGTCAGGACGCCTCCTTCAC
*PF2332*	SAMN04488487-2332	402	15	*Bam*H Ι *Xoh* Ι	F: AGCGGATCCATGGCCTTCTCAACCCAA
					R: GGACTCGAGCTAACGAGCGGTAATCAC
*PF2361*	SAMN04488487-2361	1350	50	*Bam*H Ι *Xoh* Ι	F: AGGGGATCCATGAAGACGTTAATAAGC
					R: GGCCTCGAGCTACCAGAGTGGAACGAC
*PF2405*	SAMN04488487-2405	1287	47	*Bam*H Ι *Xoh* Ι	F: AGGGGATCCATGTTGAAGCAACGGATG
					R: TTGCTCGAGTTAGAAAACGTTGATCGG
*LolA*	SAMN04488487-2577	591	22	*Bam*H Ι *Xoh* Ι	F: ACAGGATCCATGAGAGTTTTAATACTG
					R: CCGCTCGAGTCACTGGGCAAAGTCGTG
*PF2855*	SAMN04488487-2855	1320	48	*Bam*H Ι *Xoh* Ι	F: CTTGGATCCATGAATATTGCTTACTAC
					R: GTTCTCGAGTCAAAGTGCTACTAGCGG
*PF2971*	SAMN04488487-2971	1314	48	*Bam*H Ι *Xoh* Ι	F: TGGGGATCCATGACGGGCGACTCGCAACT
					R: CCGCTCGAGCTACCAGAGGTAACGATATC
*PF3127*	SAMN04488487-3127	1395	51	*Bam*H Ι *Xoh* Ι	F: TGGGGATCCATGAAACTGAGCAGCAAA
					R: GGTCTCGAGTTAAAGAATGTTGAACGGGA
*PF3231*	SAMN04488487-3231	777	29	*Bam*H Ι *Xoh* Ι	F: TGGGGATCCATGTTCACTTCGCGTCGTC
					R: GGTCTCGAGTTACTGGTACTGCTGGTA
*PF3256*	SAMN04488487-3256	1323	49	*Bam*H Ι *Xoh* Ι	F: ACCGGATCCGTGAAGAACTCGCTGACG
					R: AAGCTCGAGTTAGAACACGTTGATCGGG
*PF3269*	SAMN04488487-3269	1512	55	*Bam*H Ι *Xoh* Ι	F: ACAGGATCCGTGCCGCGTCGCATCAACA
					R: CCTCTCGAGTCAGTGATCAAACACGGCA
*PF4591*	SAMN04488487-4591	1317	48	*Bam*H Ι *Xoh* Ι	F: TTGGGATCCATGAATCACACCACTTGC
					R: ATTCTCGAGTTACATCAGCGGGATGCT
*PF4616*	SAMN04488487-4616	1410	52	*Bam*H Ι *Xoh* Ι	F: AGAGGATCCATGAAAATTTCTACACTG
					R: AGGCTCGAGTTAGAAGATCGCGTAGGT
*OmpV*	SAMN04488487-5523	792	29	*Bam*H Ι *Xoh* Ι	F: GACGGATCCATGCGCCCAAAAAACCTGT
					R: GTCCTCGAGTCAGAACGTGTATTCCAC

**Table 2:** Passive immunoprotection of OMP mouse sera in *C. auratus*.

Protein Name	Titer	Immunity (μL)	Nos	Survival No.	Death No.	ADR (%)	RPS (%)
PF2253	1:12,800	40	15	12	3	20.0	78.6 **
PF4616	1:3200	80	15	12	3	20.0	78.6 **
SurA	1:102,400	10	15	11	4	26.7	71.4 **
PF1798	1:51,200	20	15	11	4	26.7	71.4 **
PF0542	1:25,600	20	15	10	5	33.3	64.3 **
ExbB	1:12,800	40	15	9	6	40.0	57.1 *
PF1756	1:51,200	20	15	8	7	46.7	50 *
PF1068	1:3200	80	15	7	8	53.3	42.9 *
PF1380	1:25,600	20	15	7	8	53.3	42.9 *
SlyB	1:12,800	40	15	4	11	73.3	21.4
PF2361	1:3200	80	15	4	11	73.3	21.4
PF2405	1:204,800	10	15	4	11	73.3	21.4
PF3256	1:6400	40	15	4	11	73.3	21.4
PF3127	1:6400	40	15	3	12	80.0	14.3
PF3269	1:6400	40	15	2	13	86.7	7.1
PF2855	1:3200	80	15	1	14	93.3	0
Control (blank serum)	--	100	15	1	14	93.3	--

ADR: accumulated death rate. RPS: relative percent survival; RPS (%) = [1 − (% OMP sera mortality/% control serum mortality)] × 100. * *p* < 0.05 and ** *p* < 0.01 (compared with the control).

## Data Availability

The data presented in this study are available on request from the corresponding author.

## Supplementary Materials

The following supporting information can be downloaded at: https://www.mdpi.com/article/10.3390/life16091493/s1, Table S1: Primers used for the qRT-PCR; Table S2: Passive protective abilities of the entire OMP mouse serum to *P. fluorescens* and *A. hydrophila* in *C. auratus*. Table S3: Passive cross-protective abilities of the five OMP mouse sera to *A. hydrophila* in *C. auratus*; Table S4: Active protective abilities of the five OMPs to *P. fluorescens* in *C. auratus*; Table S5: Active cross-protective abilities of the five OMPs to *A. hydrophila* in *C. auratus*; Table S6: Similarity comparison of the three protective antigen protein sequences with homologous proteins from *A. hydrophila*; Raw data.
