# Supplementary material for: The Vaccine Candidates Against Major Fish Pathogens (Pseudomonas fluorescens and Aeromonas hydrophila) from the Outer Membrane Proteins of P. fluorescens in Fish
Source: Life (Basel). 2026 Sep 6;16(9):1493. doi: 10.3390/life16091493 (PMC13608734; doi:10.3390/life16091493)
Supplement: Supplementary file 1 [file life-16-01493-s001.zip › Supplementary Table S2.pdf]

Supplementary Table S2. Passive protective abilities of the entire OMP mouse serum to *P. fluorescens* and *A. hydrophila* in *C. auratus*.

Bacteria	Group	Nos	Survival (No.)	Death (No.)	ADR (%)	RPS (%)	*P* value
P. fluorescens	Entire OMP serum	15	10	5	66.7	61.5**	0.003
	Control (blank serum)	15	2	13	13.3	--	--
A. hydrophila	Entire OMP serum	15	9	6	60.0	57.1**	0.002
	Control (blank serum)	15	1	14	6.7	--	--

ADR: accumulated death rate. RPS: relative percent survival; RPS (%) = [1 − (% the entire OMP serum mortality/% control serum mortality)] × 100. **$p < 0.01$ (compared with the control).
