# Supplementary material for: The Vaccine Candidates Against Major Fish Pathogens (Pseudomonas fluorescens and Aeromonas hydrophila) from the Outer Membrane Proteins of P. fluorescens in Fish
Source: Life (Basel). 2026 Sep 6;16(9):1493. doi: 10.3390/life16091493 (PMC13608734; doi:10.3390/life16091493)
Supplement: Supplementary file 1 [file life-16-01493-s001.zip › Supplementary Table S3.pdf]

Supplementary Table S3. Passive cross-protective abilities of the five OMP mouse sera to *A. hydrophila* in *C. auratus*.

Name	Nos	Survival (No.)	Death (No.)	ADR (%)	RPS (%)	*P* value
PF0542	15	10	5	33.33	61.5**	0.003
SurA	15	11	4	26.67	69.2**	0.0009
PF1798	15	9	6	40.00	53.8**	0.008
PF2253	15	9	6	40.00	53.8**	0.008
PF4616	15	9	6	40.00	53.8**	0.008
Control (blank serum)	15	2	13	86.67	--	--

ADR: accumulated death rate. RPS: relative percent survival; RPS (%) = [1 − (% OMP sera mortality/% control serum mortality)] × 100. $^{**}p < 0.01$ (compared with the control).
