# Supplementary material for: The Vaccine Candidates Against Major Fish Pathogens (Pseudomonas fluorescens and Aeromonas hydrophila) from the Outer Membrane Proteins of P. fluorescens in Fish
Source: Life (Basel). 2026 Sep 6;16(9):1493. doi: 10.3390/life16091493 (PMC13608734; doi:10.3390/life16091493)
Supplement: Supplementary file 1 [file life-16-01493-s001.zip › Supplementary Table S4.pdf]

Supplementary Table S4. Active protective abilities of the five OMPs to *P. fluorescens* in *C. auratus*.

Name	Nos	Survival (No.)	Death (No.)	ADR (%)	RPS (%)	*P* value
PF0542	15	12	3	20	66.7*	0.025
SurA	15	13	2	13.33	77.8**	0.008
PF1798	15	13	2	13.33	77.8**	0.008
PF2253	15	12	3	20	66.7*	0.025
PF4616	15	13	2	13.33	77.8**	0.008
Control	15	6	9	60	--	--

ADR: accumulated death rate. RPS: relative percent survival; RPS (%) = [1 − (% OMP mortality/% control mortality)] × 100. The control group represents the crucian carp are immunized with the vector tag protein of pET-32a plasmid. * $p < 0.05$ and ** $p < 0.01$ (compared with the control).
