# Supplementary material for: The Vaccine Candidates Against Major Fish Pathogens (Pseudomonas fluorescens and Aeromonas hydrophila) from the Outer Membrane Proteins of P. fluorescens in Fish
Source: Life (Basel). 2026 Sep 6;16(9):1493. doi: 10.3390/life16091493 (PMC13608734; doi:10.3390/life16091493)
Supplement: Supplementary file 1 [file life-16-01493-s001.zip › Supplementary Table S6.pdf]

Supplementary Table S6. Similarity comparison of the three protective antigen protein sequences with homologous proteins from *A. hydrophila*.

Protein	Homologous protein name	NCBI Accession	Percentage identity (%)	Query coverage (%)	E value	Total score
PF0542	YfiB	WP_414627663.1	36.18	54	1e-15	73.2
	OmpA family protein	MDX2126120.1	37.29	43	2e-15	71.2
SurA	SurA	HHQ4942334.1	40.54	74	3e-89	275
	Peptidylprolyl isomerase	WP_408823311.1	36.08	22	2e-10	63.2
PF2253	Efflux transporter outer membrane subunit	WP_241872487.1	33.04	95	9e-57	196
	OprM	AXL05114.1	28.27	79	5e-42	154
	TolC family protein	HAT2528630.1	24.15	60	9e-14	72.4
